# Bacterial Communities Across the Production Chain of a Pacific Oyster (*Magallana gigas*) Hatchery During a Larval Mortality Event

**DOI:** 10.3390/pathogens15080830

**Published:** 2026-08-07

**Authors:** Xiang Zhang, Tao Yu, Zu-De Song, Bo-Wen Huang, Yu-Dong Zheng, Chong-Ming Wang, Chang-Ming Bai

**Affiliations:** 1 State Key Laboratory of Agricultural Products Safety, Ningbo University, Ningbo 315211, China; zhxiang1997@126.com; 2 State Key Laboratory of Mariculture Biobreeding and Sustainable Goods, Key Laboratory of Maricultural Organism Disease Control, Ministry of Agriculture, Yellow Sea Fisheries Research Institute, Chinese Academy of Fishery Sciences, Qingdao 266071, China; huangbw@ysfri.ac.cn (B.-W.H.); zhengyudong032@163.com (Y.-D.Z.); wangcm@ysfri.ac.cn (C.-M.W.); 3 Changdao Enhancement and Experiment Station, Chinese Academy of Fishery Sciences, Changdao 265800, China; cdyutao@126.com; 4 Changdao Marine Ecological Civilization Comprehensive Experimental Area Marine Economy Promotion Center, Changdao 265899, China; cdxscyjs@163.com; 5 Laboratory for Marine Fisheries Science and Food Production Processes, Qingdao National Laboratory for Marine Science and Technology, Qingdao 266237, China; 6 Shandong Technology Innovation Center of Oyster Seed Industry, Qingdao 266105, China

**Keywords:** *Magallana gigas*, larval microbiome, 16S rRNA amplicon sequencing, hatchery disease, water treatment

## Abstract

Bacterial disease is a major constraint on *Magallana gigas* larval production, yet hatchery microbiology has been characterised almost exclusively through the lens of *Vibrio*, and rarely across the whole production chain. We tracked bacterial communities throughout an entire larval rearing cycle at a commercial hatchery in northern China. A total of 78 samples were collected from the full production chain, from water intake to larvae. Bacterial communities were characterised using 16S rRNA (V4–V5) amplicon sequencing and culture-based isolation, with larvae sampled from three replicate tanks at six developmental stages. A protracted mortality event began at the D-veliger stage, with cumulative losses of roughly 40% before sinking ceased; ostreid herpesvirus 1 was not detected. The only taxon that rose above its healthy baseline during larval mortality was a single undescribed a single undescribed amplicon sequence variant (ASV) of the family Cryomorphaceae. Its relative abundance increased to a mean of 41% (with a tank-to-tank range of 33.7–51.6%) before disappearing once the mortality event concluded. No described species exceeds 92.2% 16S rRNA gene sequence identity to it, whereas its closest environmental relatives (97–98%) are, without exception, uncultured bacteria associated with marine invertebrates. It was an order of magnitude more abundant in larvae than in the surrounding water. *Vibrio* rose transiently at the onset of mortality but fell below its healthy-stage abundance while larvae were still dying. *Tenacibaculum*, by contrast, was the dominant genus of the rearing water yet was never recovered on Vibrio-selective medium and showed no association with mortality. Chlorination of the feed-room supply removed *Tenacibaculum* while leaving *Vibrio* uncontrolled. These findings reveal the limitations of the Vibrio-targeted, culture-based paradigm that has long dominated hatchery microbiology, as it overlooks both prevailing water-column organisms and mortality-linked taxa. Our results highlight the need for whole-community, culture-independent surveillance to better understand larval disease.

## 1. Introduction

The Pacific oyster (*Magallana gigas*, syn. *Crassostrea gigas*) is among the world’s most commercially important bivalves, with global aquaculture production exceeding 7 million tonnes annually [1]. Production depends on hatchery larvae, and this is the highest-risk phase of the cycle. Bacterial disease can eliminate entire cohorts within 24–48 h [2,3]. Subacute mortality events, in which a substantial fraction of the cohort is lost over several days, cause recurrent losses that remain poorly understood [4,5].

Research on bivalve hatchery disease has been dominated by the genus *Vibrio*, and with reason: several *Vibrio* species reproducibly kill bivalve larvae in challenge experiments, and hatchery epizootics attributed to vibriosis have been documented for four decades [3,6,7,8,9,10,11,12]. Targeted interventions such as phage therapy have been developed against the principal etiologies [13]. This focus is partly methodological: *Vibrio* grows rapidly on standard and selective media, whereas other marine opportunists are systematically under-recovered by culture. A notable example is the genus *Tenacibaculum*: these slow-growing, marine-medium-dependent gliding bacteria include serious fish pathogens [14] yet are excluded by design from Vibrio-selective media. Selective culture cannot reveal the organisms it suppresses [15]. Whether organisms other than *Vibrio* contribute to larval mortality under commercial conditions therefore remains unresolved, and culture-dependent approaches alone cannot resolve it.

The bacterial community of bivalve larvae changes markedly across development and participates in nutrient cycling, immune priming and competitive exclusion [7,16,17]. Healthy and moribund larvae harbour distinct communities [8,18], shaped by extrinsic factors such as rearing water, the microalgal feed supply, broodstock, and physicochemical conditions [19,20,21]. Most studies, however, have sampled only a subset of these compartments or followed communities over short periods under controlled conditions [22]. Longitudinal sampling across the entire production chain—from seawater intake through treatment, broodstock, feed production, and rearing tanks to the larvae—remains scarce; yet it is essential for identifying the sources and transmission routes of candidate pathogens.

A further consequence of the Vibrio-centred literature is that abundant but unnamed lineages attract little attention. Amplicon surveys of bivalve larvae routinely report substantial fractions of sequences that cannot be assigned to a described genus (for instance, ~27% in one Pacific oyster larval survey) [23], and these are commonly aggregated into an “unclassified” category and set aside.

Commercial Pacific oyster hatcheries in northern China operate from late spring through summer [24], when rising sea temperatures favour opportunistic bacteria [25,26]. Raw coastal seawater typically undergoes a two-stage physical treatment (sedimentation followed by sand filtration) before entering larval tanks, whereas chemical disinfection (sodium hypochlorite, followed by thiosulfate neutralisation) is reserved for the microalgae culture facility. Neither treatment has been evaluated at the whole-community level. In particular, it remains unknown whether chlorination suppresses all opportunistic bacteria equally or instead selects for tolerant lineages, and what subsequently proliferates in the treated water.

This study was prompted by recurrent larval mortality during commercial production of hatchery larvae in China. We profiled bacterial communities across the entire production chain using 16S rRNA gene amplicon sequencing, complemented by culture-based isolation and near-full-length 16S rRNA gene identification of isolates, while qPCR excluded ostreid herpesvirus 1 (OsHV-1). Specifically, we examined: (i) developmental and mortality-associated changes in the larval microbiota; (ii) taxa linked to mortality; (iii) potential sources of candidate organisms; and (iv) the effect of feed-room chlorination on their abundance.

## 2. Materials and Methods

### 2.1. Study Site and Production Overview

The study was conducted at a commercial *Magallana gigas* hatchery in Shandong Province, northern China (Yellow Sea coast) in 2021. Coastal seawater was drawn from an open intake, passed sequentially through a settling pond (~12 h retention) and a sand filter before being delivered to 20,000 L concrete larval rearing tanks. Neither ultraviolet irradiation nor ozone treatment was applied to the seawater. In a separate microalgae culture facility, sand-filtered water was disinfected with sodium hypochlorite and subsequently neutralised with sodium thiosulfate before use in microalgal culture.

Broodstock were conditioned and spawned on 26 June 2021. Fertilised eggs were stocked at approximately 5 eggs·mL^−1^. Water exchanges (two-thirds) were performed every 24 h. Rearing water temperature was maintained at 25 ± 1 °C and salinity at 28‰ throughout. Larvae were fed *Isochrysis galbana* and *Chaetoceros muelleri* twice daily, at a combined daily ration of approximately 4 × 10^4^ cells·mL^−1^, doubled from the umbo stage onwards. Sampling was conducted at the D-veliger stage on 29 June. Beginning on 30 June, heavy larval sinking and accumulation on tank bottoms were observed, with a peak on 30 June and 2 July. Affected larvae swam sluggishly and showed reduced activity before settling to the bottom; no other externally visible abnormalities were observed. Mortality was assessed solely by visual inspection of larval density. Quantitative enumeration and histological examination were not performed: the study was conducted in a working commercial hatchery during a production run, the mortality was not anticipated, and neither routine practice at the facility nor the conditions on site permitted larvae to be removed for counting or fixed for histology without disrupting production. This limitation is stated in Section 4.7 and does not affect the sequence-based analyses on which all conclusions here rest. Surviving larvae developed through the umbo stages to the eyed pediveliger.

Larvae analysed in this study were collected from three rearing tanks that received no exogenous microbial additions and into which no larvae were transferred at any point during the production cycle. These three tanks constitute the biological replicates (*n* = 3) for all larval analyses.

### 2.2. Sample Collection

**Figure 1:**
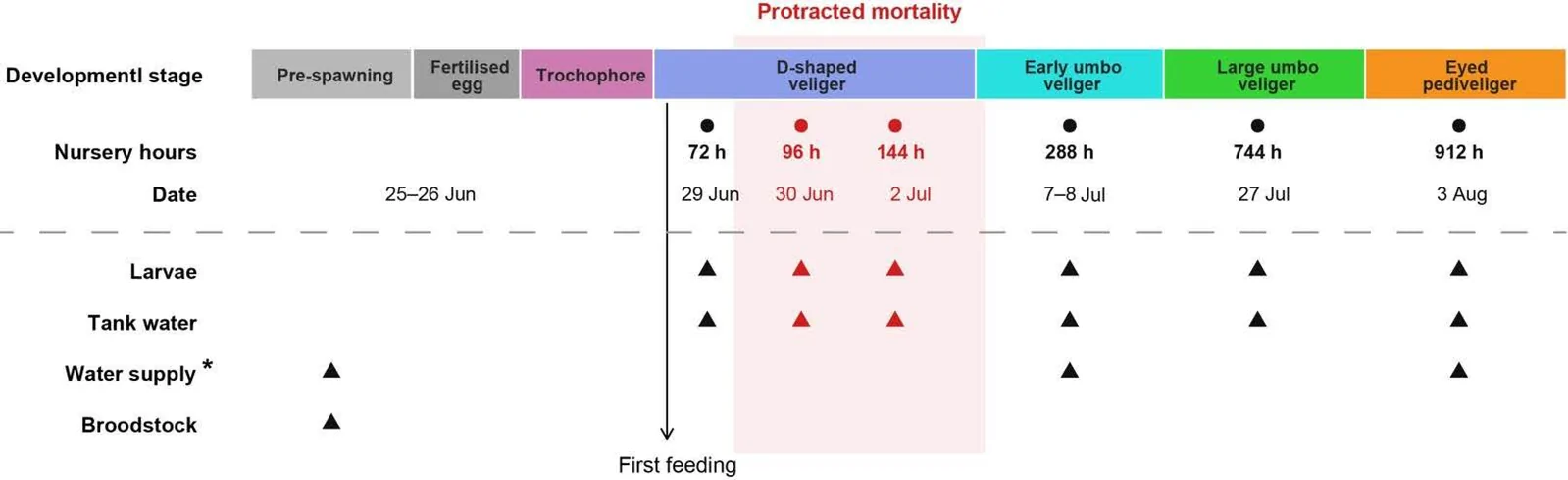
Whole-chain sampling design. Samples were collected across the hatchery production chain and over the course of larval development. Coloured bars denote developmental stages; filled triangles mark samples taken for 16S rRNA gene sequencing (three replicates each). The dashed horizontal line separates the developmental timeline (above) from the sampling record (below). Red symbols and dates denote time points falling within the mortality event; black symbols denote all other time points. Larvae and their rearing-tank water were sampled at six stages, from the D-veliger stage (72 h post-fertilisation) to the eyed pediveliger (912 h). A protracted mortality event (shaded) was sampled at two time points, day 1 (30 June) and day 3 (2 July), when larvae that had settled to the tank bottom were collected. Broodstock (pond and spawning-pond water, and adult tissue) and the incoming water-supply chain were sampled before spawning, with the water supply partly re-sampled at later stages (see the asterisked note below). Developmental time is expressed as hours post-fertilisation. * Full intake chain (seawater, settling pond, sand-filtered water, and disinfected [EQ] and chlorinated [PEC] feed-room water) sampled at pre-spawning; settling pond, sand-filtered water and EQ were resampled at the early-umbo and eyed stages.

A total of 78 samples were collected, representing triplicate samples at each sampling point across the entire system (Figure 1, Table S1). Water supply chain samples consisted of the open sea intake (25 June) and settling pond and sand filter outflows (25 June, 7 July, 3 August). Broodstock and spawning samples comprised broodstock pond water, spawning-pond water, and broodstock tissue (26 June). Feed-room samples consisted of water collected after chlorination and neutralisation (EQ; 25 June, 7 July, 3 August) and water from a storage tank after chlorination but before neutralisation (PEC; 25 June only); disinfected water was stored for approximately one to two days before use, although the exact residence time was not recorded. Larval samples were collected from the same three tanks at six time points: healthy D-veliger (29 June), day 1 of mortality (30 June), day 3 of mortality (2 July), early umbo (8 July), large umbo (27 July), and eyed pediveliger (3 August). At the two mortality time points, moribund larvae that had settled to the tank bottom were sampled. Larvae were retained on a clean 300-mesh (~48 µm) sieve, transferred to microcentrifuge tubes, and rinsed twice with sterile seawater by gentle pipetting before storage at −80 °C. Rearing-tank water was collected from the same three tanks at each of the six larval time points. All water samples (1 L) were filtered onto 0.22 µm polycarbonate membranes (Merck Millipore, Burlington, MA, USA), and all samples were stored at −80 °C until DNA extraction. Because larvae and rearing-tank water were sampled from the same three tanks at every time point, developmental stage constitutes a within-tank (repeated-measures) factor, and analyses were structured accordingly (Section 2.7).

### 2.3. Bacterial Isolation and Identification

Bacterial isolation was performed from seawater, settling pond water, broodstock pond water, broodstock tissue, feed-room water, healthy larvae, and moribund larvae collected during the mortality event. For larval samples, approximately 50 mg of tissue was homogenised with 500 µL of sterile seawater on ice using a sterile disposable pestle (Axygen^®^ PES-15-B-SI; Corning Inc., Corning, NY, USA). After mixing, 50 µL of the supernatant was spread onto 2216E marine agar plates (Solarbio, cat. no. LA0340; Beijing Solarbio Science & Technology Co., Ltd., Beijing, China) and TCBS agar plates (Thiosulfate Citrate Bile Salts Sucrose Agar, cat. no. HB4130; Qingdao Hope Bio-Technology Co., Ltd., Qingdao, China). For water samples, 100 µL of each sample was directly spread onto the same two types of agar plates. All plates were incubated at 28 °C for 24–48 h and examined for bacterial growth.

After incubation, colonies were categorised according to their size, shape, and colour. For each sample, colonies of each type were counted, and dominant types were defined as those constituting at least 30% of all colonies. A representative colony of each dominant type was picked with a sterile loop and streaked onto fresh plates for purification. Purified isolates were incubated in 1 mL of 2216E liquid medium (Solarbio, cat. no. LA0341) at 28 °C overnight, and 600 µL of liquid culture containing 40% glycerol was stored at −80 °C.

For identification, purified isolates were sequenced by the Sanger method across the near-full-length 16S rRNA gene using bacterial primers 27F (5′-AGAGTTTGATCMTGGCTCAG-3′) and 1492R (5′-TACGGYTACCTTGTTACGACTT-3′), which yield a product of approximately 1500 bp. Reactions (50 µL) contained 25 µL of Premix Taq™ (Takara Bio Inc., Dalian, China), 1 µL of each primer (10 µM), 2 µL of template DNA and nuclease-free water to volume. Thermal cycling comprised an initial denaturation at 95 °C for 5 min; 30 cycles of 95 °C for 30 s, 55 °C for 30 s and 72 °C for 90 s; and a final extension at 72 °C for 10 min. Sequences were compared against the NCBI 16S rRNA reference database by BLASTn (NCBI, Bethesda, MD, USA; accessed 8 July 2025); the closest type strain and percentage identity are presented in Table S2.

### 2.4. Screening for Ostreid Herpesvirus 1 (OsHV-1)

All larval samples (*n* = 18; three tanks × six developmental stages, including moribund larvae from both mortality samplings) were screened for OsHV-1 by real-time quantitative PCR targeting the viral B region, using primers BF (5′-GTCGCATCTTTGGATTTAACAA-3′) and B4 (5′-ACTGGGATCCGACTGACAAC-3′) with the hydrolysis probe B-P (5′-6FAM-TGCCCCTGTCATCTTGAGGTATAGACAATC-BHQ1-3′) [27]. Reactions (25 µL) contained 12.5 µL of 2× FastStart Essential DNA Probes Master (Roche Diagnostics GmbH, Mannheim, Germany), 0.5 µL of each primer (20 µM), 0.5 µL of probe (10 µM), 2 µL of template DNA and 9 µL of water. Amplification was performed on a Bio-Rad CFX Connect Real-Time system (Bio-Rad Laboratories, Hercules, CA, USA) with one cycle of 95 °C for 10 min followed by 40 cycles of 95 °C for 10 s and 60 °C for 30 s. The template was the same genomic DNA used for 16S rRNA amplicon sequencing. Each run included a plasmid standard carrying the target amplicon as a positive control, together with no-template controls. Samples were scored as negative when no amplification was detected within 40 cycles. No host-gene internal control was included, and PCR inhibition therefore cannot be formally excluded; however, all templates yielded abundant bacterial 16S amplicons (>42,000 quality-filtered reads per sample), confirming that they were amplifiable.

### 2.5. DNA Extraction and 16S rRNA Gene Amplicon Sequencing

DNA extraction, PCR, library preparation and sequencing were performed by a commercial provider (Novogene Co., Ltd., Beijing, China). Genomic DNA was extracted by the CTAB/SDS method and assessed by agarose gel electrophoresis. The V4–V5 hypervariable region of the bacterial 16S rRNA gene was amplified with barcoded primers 515F (5′-GTGCCAGCMGCCGCGGTAA-3′) and 907R (5′-CCGTCAATTCMTTTRAGTTT-3′). The primers yield a product of approximately 400 bp. Reactions (30 µL) contained 15 µL of Phusion^®^ High-Fidelity PCR Master Mix with GC Buffer (New England Biolabs, Ipswich, MA, USA), 2 µM of each primer, and approximately 10 ng of template DNA. Thermal cycling comprised an initial denaturation at 98 °C for 1 min; 30 cycles of 98 °C for 10 s, 50 °C for 30 s and 72 °C for 30 s; and a final extension at 72 °C for 5 min. Amplicons were purified, pooled in equimolar amounts, and sequenced on an Illumina NovaSeq 6000 platform (paired-end, 2 × 250 bp). Reads were merged and primer sequences removed by the provider; merged reads averaged 372 bp and were the input for all downstream processing.

### 2.6. Bioinformatic Processing

Processing followed the EasyAmplicon pipeline [28], using VSEARCH v2.30.1 [29] and USEARCH v10.0.240 [30]. Merged reads were quality-filtered (vsearch --fastx_filter, maximum expected error rate 0.01; no primer stripping required), dereplicated (--derep_fulllength --minuniquesize 10; 30,420 unique sequences from 5,513,207 reads), and denoised with UNOISE3 [31] (-minsize 10), yielding 4717 amplicon sequence variants (ASVs). Reference-based chimaera removal (--uchime_ref) against the Ribosomal Database Project (RDP) 16S training set v18 left 4537 ASVs. Quality-filtered reads were mapped to ASV representatives (--usearch_global --id 0.97; 97.96% of unique query sequences mapped). Taxonomy was assigned with SINTAX against RDP v18 [32] at two thresholds: a low-confidence assignment (--sintax_cutoff 0.1) and a conservative assignment (--sintax_cutoff 0.8). The conservative assignment was used for all taxonomic summaries and figures. ASVs assigned to chloroplasts, mitochondria or non-bacterial lineages were removed (4356 ASVs remaining).

Given the commercial processing of samples without negative controls, empirical identification of contaminants was not feasible. ASVs assigned, at any confidence in the low-threshold annotation, to genera repeatedly reported as sequencing contaminants [33], spanning reagent/kitome, human-associated, and freshwater/soil taxa, were removed manually (163 ASVs, 2.42% of reads; full genus list, SINTAX confidences and categories in Table S4). *Pseudomonas* was retained: it contains genuine marine members and was most abundant in high-biomass natural seawater rather than in low-biomass samples, a distribution inconsistent with reagent contamination [33]. *Vibrio*, *Tenacibaculum* and *Thiomicrorhabdus* were not among the removed taxa. A further 63 ASVs assigned to mammalian-gut anaerobes (*Alistipes*, Lachnospiraceae; 0.276% of reads, sporadically distributed) were judged to reflect low-level cross-contamination during multiplexed sequencing rather than reagent contamination; they were retained, and their exclusion does not alter any result reported here (Supplementary Methods).

**Figure 2:**
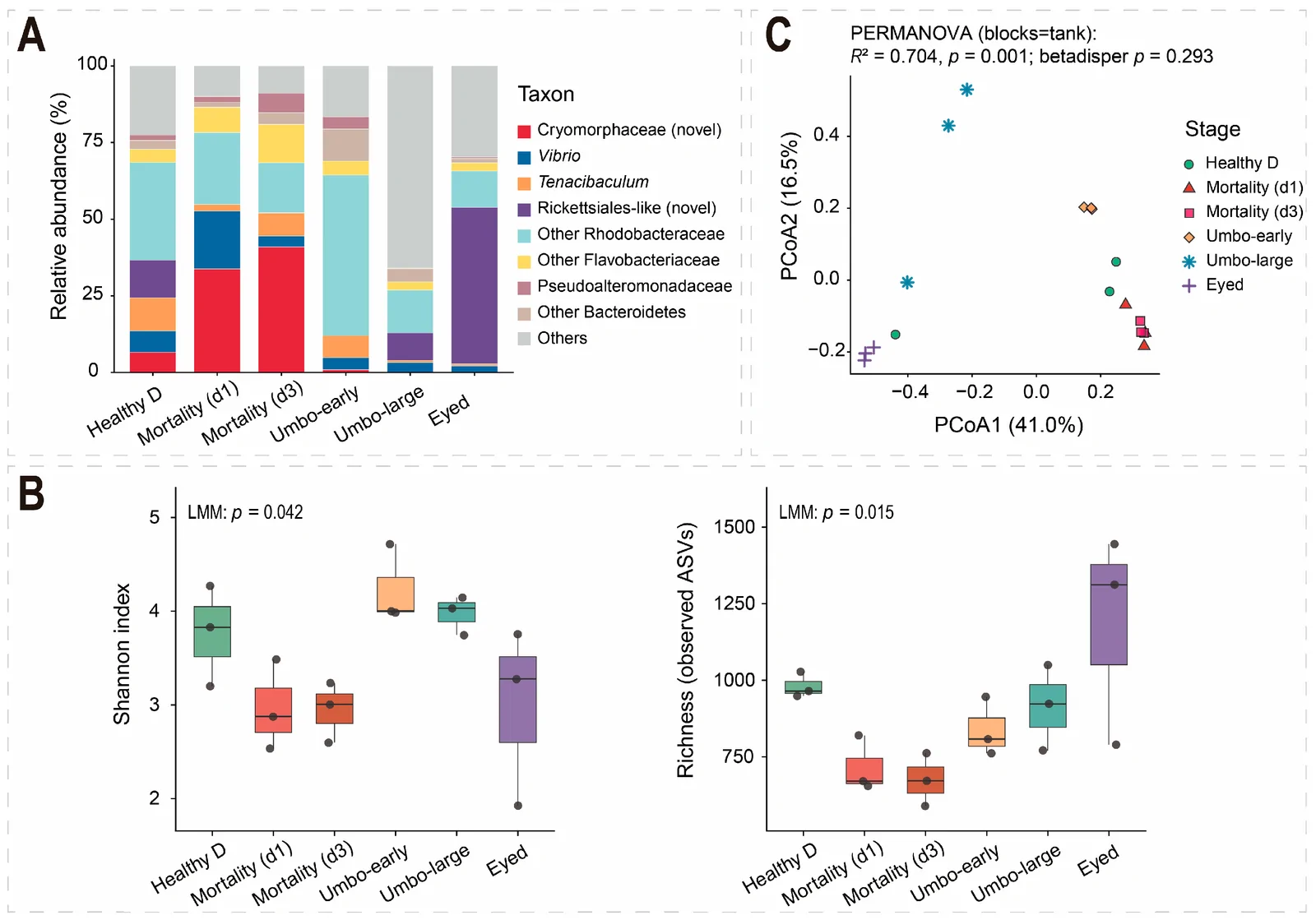
Larval microbiota across development. (**A**) Relative abundance of key taxa and higher-level groups at six developmental stages (mean of three replicate tanks). (**B**) Shannon index and observed ASV richness (boxes, median and median and interquartile range (IQR); points, individual tanks). Diversity differed among stages overall (linear mixed model with tank as random effect: richness *p* = 0.015; Shannon *p* = 0.042), but no healthy-versus-mortality contrast was significant after Tukey adjustment (all *p* > 0.18); reduced diversity at the mortality samplings is reported as a trend. (**C**) Bray–Curtis PCoA (permutational multivariate analysis of variance (PERMANOVA), permutations restricted within tank: R^2^ = 0.704, *p* = 0.001; betadisper *p* = 0.293); ellipses, 68% confidence regions. Mortality samplings are day 1 (30 June) and day 3 (2 July) of a single protracted event.

The feature table was rarefied to 40,000 reads per sample (seed = 1). All 78 samples exceeded this depth (minimum 42,377) and were retained, yielding 4191 ASVs (Figure S1). Genus-level abundances of *Vibrio*, *Tenacibaculum*, and *Thiomicrorhabdus* were obtained by summing all ASVs matching the genus in the low-threshold (0.1) annotation, thereby avoiding the under-counting that confidence filtering causes by collapsing low-confidence assignments. *Photobacterium* (4 ASVs, 0.008% of reads) was pooled with *Vibrio*, and the group is referred to as *Vibrio* throughout. Family-level composition (Figure 2A and Figure S2) was derived from the confidence-filtered table; genus- and family-level percentages are therefore not directly comparable.

Representative sequences of the two dominant unclassified ASVs were queried by BLASTn (NCBI web service, accessed 8 July 2025) against (i) the NCBI 16S rRNA reference database (type strains only), to identify the closest described species, and (ii) the full nucleotide collection (nr/nt, megablast), to identify the closest environmental sequences and their reported sources. Genus- and species-level boundaries were taken as 94.5% and 98.7% 16S identity, respectively [34]. Query coverage is reported alongside identity throughout (Table S3). Novelty was assessed on the V4–V5 region (~370 bp after primer trimming), which affords greater phylogenetic resolution than the V4 region alone. The 16S rRNA gene sequences of ASV_3 and ASV_4 were aligned with reference sequences of representative Rickettsiales, Cryomorphaceae and an outgroup (*Escherichia coli*) using MAFFT (v7.520), trimmed of poorly aligned positions with trimAl (v1.4.1) (gap threshold 0.5), and used to infer a maximum-likelihood tree in IQ-TREE (v2.2.2.6) (best-fit model TIM3 + F + G4 by BIC; 1000 ultrafast bootstrap replicates).

### 2.7. Statistical Analysis

All analyses were performed in R 4.5.2 (RStudio 2025.09.2). Alpha diversity indices were computed from the rarefied table with vegan (v2.7.3) [35]. The effect of developmental stage on each response was tested by linear mixed models with stage as a fixed effect and tank as a random intercept (lmerTest (v3.2.1); [36,37]), with Satterthwaite approximation for denominator degrees of freedom. Relative abundances were arcsine square-root transformed before modelling; diversity indices were analysed untransformed. Pairwise contrasts among the six stages were obtained with emmeans (v2.0.3) [38] and adjusted by Tukey’s method (15 contrasts). The comparison between moribund larvae and healthy D-veligers with respect to *Vibrio* relative abundance was prespecified as the primary contrast and is presented without adjustment for multiple testing. All secondary pairwise comparisons (*n* = 15) were performed with Tukey’s adjustment. For two responses (*Vibrio*, ASV_3) the random-effect variance was estimated at zero (singular fit), indicating no detectable between-tank clustering; these models reduce to fixed-effects equivalents and are reported as such. Friedman tests (stage as treatment, tank as block) were computed as a distribution-free reference for each response and are reported alongside the mixed-model results (Table S5). Bray–Curtis dissimilarities were computed from relative abundances. Community differences among developmental stages were tested by PERMANOVA (adonis2, 999 permutations), with permutations restricted to within tanks (permute: how (blocks = tank)) to respect the repeated-measures design. Homogeneity of multivariate dispersion was assessed with betadisper and permutest. Ordination used principal coordinates analysis. Differences among water types were tested by Kruskal–Wallis, with pairwise Mann–Whitney tests where specified. EQ samples from the three months were analysed both separately and pooled; because diversity in EQ differed markedly among months, the pooled comparison is reported with this heterogeneity noted. With three replicates, some non-parametric comparisons cannot reach significance regardless of effect size (e.g., Mann–Whitney 3 vs. 3 has a minimum two-sided *p* of 0.10); we therefore emphasise effect sizes and cross-tank consistency, and avoid causal language where significance is unattainable. Mixed models, which recover more power under this design, are the primary analysis for larval responses. Figures were produced with ggplot2 (v4.0.3) [39], assembled with patchwork (v1.3.2), and finalised in Adobe Illustrator 2022.

## 3. Results

### 3.1. Sequencing Overview, Contaminant Removal, and Exclusion of OsHV-1

All 18 larval samples (three tanks × six developmental stages, including moribund larvae from the two mortality sampling time points) tested negative for OsHV-1 by qPCR, while the plasmid positive control amplified as expected. Although no host-gene internal control was included, the same DNA yielded abundant bacterial 16S rRNA gene amplicons (Section 2.4), confirming that it was amplifiable. OsHV-1 is therefore an unlikely cause of the observed mortality.

Denoising of merged reads yielded 4537 non-chimeric ASVs. After removing chloroplast, mitochondrial, and non-bacterial sequences, 4356 ASVs remained. The commercial processing of samples without negative controls precluded empirical identification of contaminants. Therefore, 163 ASVs assigned to genera repeatedly reported as reagent-, human- or freshwater-associated contaminants [33] were removed manually (Table S4); the focal genera (*Vibrio*, *Tenacibaculum*, *Thiomicrorhabdus*) were not among the removed taxa. The filtered table was rarefied to 40,000 reads per sample; all 78 samples exceeded this depth (minimum 42,377 reads) and were retained, yielding a final dataset of 4191 ASVs.

Bacterial community composition differed markedly among compartments of the hatchery system (Figure S2). The water supply (seawater, sand-filtered water, settling and spawning ponds) was dominated by SAR11, Rhodobacteraceae and Flavobacteriaceae; rearing-tank water and the broodstock pond by Rhodobacteraceae and Flavobacteriaceae; and the feed-room equilibration tank by Piscirickettsiaceae (48.2%) and Rhodobacteraceae (36.0%). The proportion of sequences that could not be assigned to a family ranged from 6.2 to 6.3% in feed-room water and from 10.0% in the broodstock pond to 20.7–25.7% across the incoming water supply, rising to 45.1% in larvae and 87.2% in broodstock tissue. Throughout, “*Vibrio*” denotes all ASVs assigned to *Vibrio* or the closely related *Photobacterium* (Vibrionaceae); *Photobacterium* was negligible (0.008% of reads) and does not affect any reported value.

### 3.2. The Larval Community Is Restructured Across Development

Developmental stage explained a large proportion of variation in larval bacterial community structure (PERMANOVA on Bray–Curtis dissimilarity, permutations restricted within tank: *R*^2^ = 0.704, *F* = 5.70, *p* = 0.001; Figure 2C). Multivariate dispersion did not differ among stages (betadisper, *p* = 0.293), indicating that this reflects displacement of group centroids rather than heterogeneity of within-group spread. Eyed pediveligers formed a tight, well-separated cluster. The first two axes explained 41.0% and 16.5% of the variance. The first two axes explained 41.0% and 16.5% of the variance (mean 1182 ASVs).

Alpha diversity varied significantly among stages (linear mixed model, LMM, with tank as random effect: richness *p* = 0.015; Shannon *p* = 0.042; Table S5; Figure 2B). No contrast between the healthy baseline and either mortality sampling was significant after Tukey adjustment; the only significant contrasts involved the eyed stage, at which observed richness reached its maximum (mean 1182 ASVs; day 1 of mortality versus eyed *p* = 0.024; day 3 of mortality versus eyed *p* = 0.014). Richness was lowest at the two mortality sampling time points (715 and 675 ASVs, versus 981 in healthy D-veligers), but neither differed significantly from the healthy baseline (*p* = 0.295 and 0.184). Shannon diversity followed a similar pattern, being lowest at the two mortality sampling time points (2.97, 2.95) and again at the eyed stage (2.99), but through different mechanisms: at the mortality time points both richness and evenness fell, whereas at the eyed stage richness was maximal and evenness was reduced by the dominance of one ASV (Section 3.6).

Compositionally (Figure 2A), healthy D-veligers harboured a mixed assemblage of Rhodobacteraceae, Flavobacteriaceae, *Vibrio* and *Tenacibaculum*. Larvae at both mortality sampling time points were dominated by a single Cryomorphaceae ASV (Section 3.3). Surviving larvae were dominated by Rhodobacteraceae at the umbo stages, and by the eyed stage the community had converged on a single divergent alphaproteobacterial ASV (Section 3.6).

### 3.3. An Undescribed Invertebrate-Associated Lineage Is the Only Taxon That Rose as Mortality Progressed

A single ASV assigned to the family Cryomorphaceae (ASV_4) was the only taxon in this dataset whose abundance rose significantly above the healthy baseline during the mortality event, and its mean abundance increased at each successive sampling as mortality progressed.

**Figure 3:**
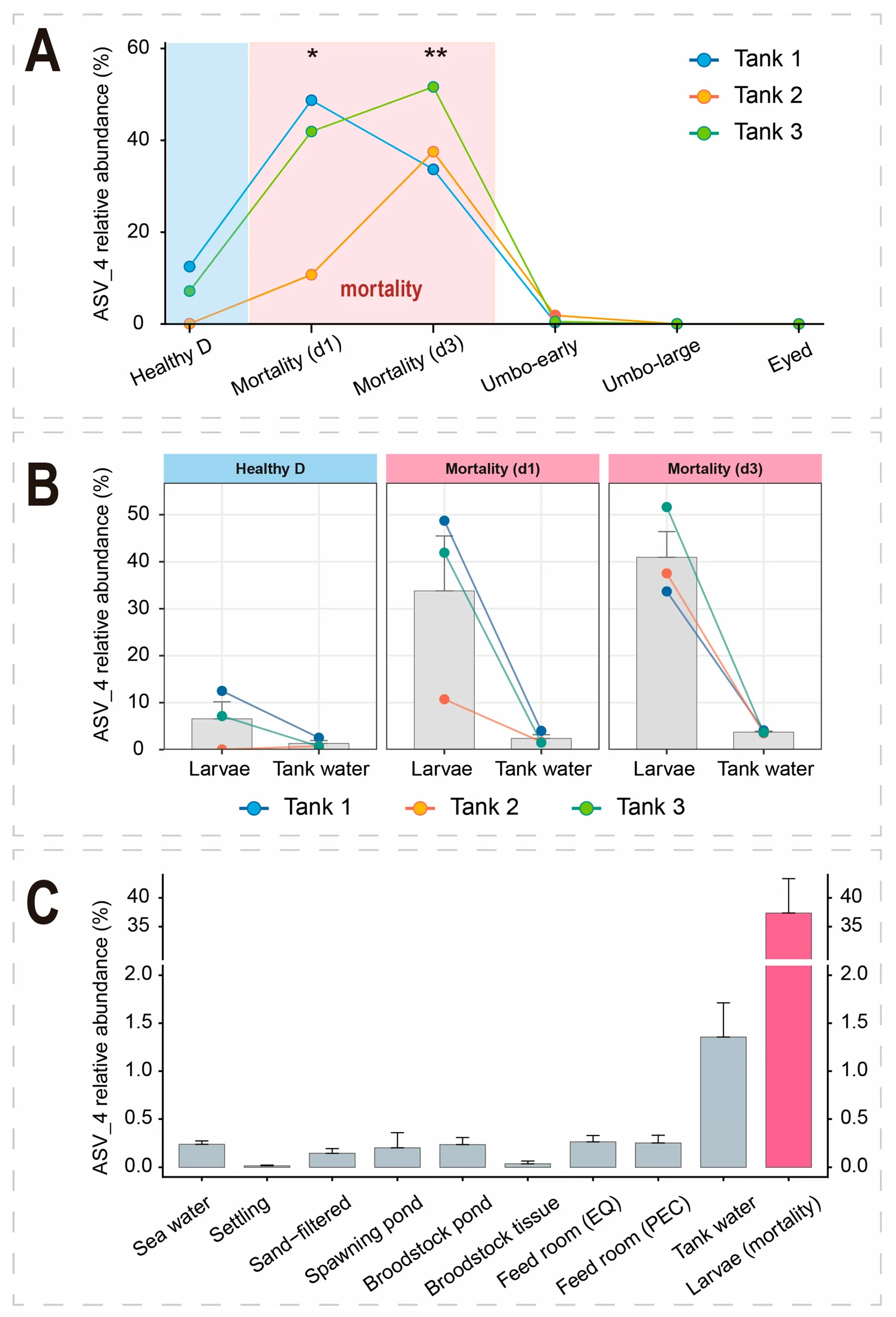
The undescribed Cryomorphaceae lineage (ASV_4) tracks larval mortality and is host-associated. (**A**) ASV_4 relative abundance in larvae across six developmental stages, shown separately for the three replicate tanks (shaded band, the protracted mortality event). Asterisks, Tukey-adjusted contrasts against healthy D-veligers (linear mixed model, tank as random effect; *, *p* < 0.05; **, *p* < 0.01). (**B**) ASV_4 in larvae versus rearing-tank water, paired by tank and day (bars, mean of three tanks; points and lines, individual tanks). (**C**) Mean (± SE) ASV_4 abundance across all sampled compartments; the pink bar denotes larvae sampled during the mortality event and grey bars denote all other compartments. Note the broken y-axis. SE, standard error.

Dynamics (Figure 3A). ASV_4 was present in healthy D-veligers (per tank: 12.5%, 0.06%, 7.2%; mean 6.6%), rose in every tank on day 1 of mortality (48.7%, 10.7%, 41.9%; mean 33.8%) and further by day 3 (33.7%, 37.5%, 51.6%; mean 41.0%), and collapsed once mortality ceased (0.9% at early umbo; ≤0.02% at all later stages). Contrasts against healthy D-veligers were significant at both mortality time points (LMM with tank as random effect, Tukey-adjusted: *p* = 0.024 and *p* = 0.006), as were contrasts between each mortality time point and every subsequent stage (*p* = 0.0004–0.004; Table S5). Its rise thus paralleled the accumulating losses, and its disappearance coincided with their cessation. Between-tank variation at the healthy stage was high (coefficient of variation, CV = 0.95), and the tank with the lowest initial abundance (tank 2) also showed the weakest bloom on day 1 (10.7% versus 41.9–48.7%) before reaching comparable levels by day 3. Whether initial load predicts the timing of proliferation cannot be tested with three tanks, and we note this as an observation only.

Host association (Figure 3B). ASV_4 was an order of magnitude more abundant in larvae than in the water of the same tank on the same day. On day 1 of mortality, it reached 41.9–48.7% in larvae of tanks 1 and 3 but only 1.5–4.0% in the corresponding tank water; on day 3, 33.7–51.6% in larvae versus 3.5–4.1% in water. It was essentially absent from the incoming water chain, the broodstock and spawning ponds, and the disinfected feed-room supply (all <0.7%; maximum 0.62% in a single feed-room sample; Figure 3C). Its proliferation therefore occurred on or within the host, not in the water column; the low levels detected in tank water are consistent with release from moribund larvae.

**Figure 4:**
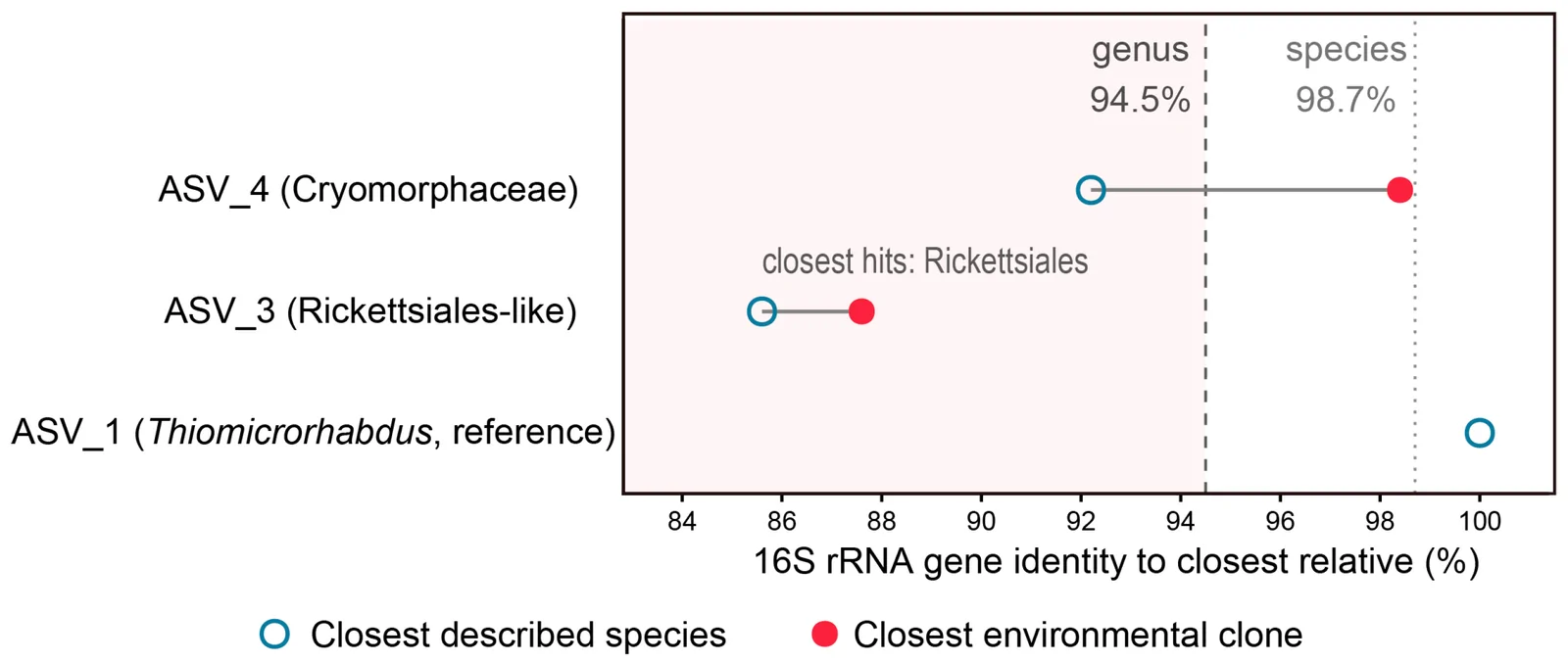
Two abundant larval ASVs fall outside all described genera. For each undescribed lineage (ASV_4, Cryomorphaceae; ASV_3, Rickettsiales-like), 16S rRNA gene identity to its closest described species (open circles) and closest environmental clone (filled circles) is shown; ASV_1 (*Thiomicrorhabdus*) is a reference taxon matching a described species at ~100%. Dashed and dotted lines indicate the 94.5% genus- and 98.7% species-level thresholds; shading indicates values below the genus threshold. For ASV_3, the single best described-species hit (*Litoreibacter arenae*) is followed immediately by numerous Rickettsiales, consistent with its assignment. Query coverage ≥ 97% for all hits; full results are provided in Table S3.

Taxonomic novelty. No isolate of this lineage was obtained. Its closest described relative is *Parvicella tangerina* (92.2% 16S identity, 100% query coverage), below the ~94.5% threshold conventionally used to delimit genera; no described species exceeded this value, and the next closest (*Croceimicrobium hydrocarbonivorans*, *Phaeocystidibacter luteus*, *Owenweeksia hongkongensis*) fell between 90.2% and 91.0%. ASV_4 therefore represents an undescribed genus-level lineage within the Cryomorphaceae (Figure 4). Its closest environmental relatives, by contrast, reach 97–98% identity, and are, without exception, epibionts, ectosymbionts or mat-forming bacteria of marine invertebrates (Table S3): uncultured sequences from an ascidian gut, a sea urchin, a galatheid crab (*Shinkaia crosnieri*), a coral, a hydrothermal shrimp (*Rimicaris exoculata*), and an ectosymbiont of the stalked barnacle *Vulcanolepas osheai* (94–99% identity; Table S3). No described species exceeds 92.2% identity, yet environmental clones reach 98.4%; the reported sources span four animal phyla (Cnidaria, Echinodermata, Chordata, Arthropoda), indicating a widely distributed but uncultivated, invertebrate-associated lineage.

### 3.4. Vibrio Rose Transiently at the Onset of Mortality but Had Subsided Before It Ended

*Vibrio* accounted for 6.9% of larval sequences in healthy D-veligers (per tank: 5.1%, 6.2%, 9.6%). On day 1 of mortality its abundance rose in every tank—to 14.7%, 28.5% and 13.3% (mean 18.8%), representing a 1.4- to 4.6-fold increase across all replicates. By day 3 it had fallen to 3.5% (1.6%, 5.8%, 3.2%), a level below that of healthy D-veligers, while larvae were still dying; it remained between 2.1% and 3.9% at all subsequent stages. Thus, the elevation in *Vibrio* abundance was transient and did not persist throughout the mortality event. Pairwise comparisons confirmed that *Vibrio* abundance on day 1 was significantly elevated relative to that on day 3, at the large umbo stage, and at the eyed stage (Tukey-adjusted *p* = 0.040, 0.012, and 0.018, respectively; Table S5). The primary prespecified contrast—the change from the healthy baseline—showed a consistent directional increase across all three replicates and was significant without multiplicity adjustment (*p* = 0.033). However, this contrast did not retain significance after Tukey adjustment for all 15 pairwise comparisons (*p* = 0.216). With three biological replicates, the design has limited power to resolve a contrast of this magnitude (Section 2.7).

*Vibrio* abundance in the water column was always below 3.1% in all compartments (highest 3.02% in broodstock pond; lowest 0.29% in rearing tanks). Its abundance in larvae on day 1 of the mortality event was roughly six times higher than in any water compartment, indicating that the transient bloom was host-associated rather than water-borne. Culture-based isolation confirmed the presence of viable *Vibrio* cells across multiple compartments. Several species were recovered from seawater, ponds, broodstock tissue, and larvae (healthy and moribund; Table S2), with *V. alginolyticus* most frequent, alongside *V. cyclitrophicus*, *V. mytili*, *V. toranzoniae*, *V. ulleungensis*, and *V. chagasii*.

### 3.5. Tenacibaculum Dominates the Rearing Water, Is Under-Recovered by Culture, and Is Unrelated to Mortality

*Tenacibaculum* was scarce throughout the incoming water supply chain and moderately enriched in the broodstock pond. In rearing-tank water, however, it was by far the most abundant genus, exceeding *Vibrio* by approximately 130-fold (Figure S4).

In larvae, *Tenacibaculum* accounted for 10.8% of sequences in healthy D-veligers (per tank: 20.6%, 2.6%, 9.2%; CV = 0.84) and fell to 2.2% on day 1 of mortality. The decline coincided with the concurrent rise of ASV_4 (to 33.8%) and *Vibrio* (to 18.8%), which together displaced 52.6% of the larval community. By day 3, *Tenacibaculum* had recovered to 7.6%, but never exceeded its healthy-stage abundance. Contrasts of day 1 of mortality against healthy D-veligers (*p* = 0.128) and day 3 (*p* = 0.988) were not significant. The significant overall stage effect (LMM *p* = 0.006) was driven entirely by the decline at later stages, where its abundance fell to 0.62% and 0.60% (Table S5).

In rearing-tank water, the community composition across the same developmental stages is shown in Figure S3. *Tenacibaculum* peaked at the healthy D-veliger stage (37.4%) and declined steadily thereafter (34.1% on 30 June; 22.2% on 2 July; 9.1% in July; 5.4% in August) (Figure S4), so its water-phase abundance did not parallel mortality. This decline may reflect daily water exchange, adsorption to tank surfaces, or seasonal succession. A sensitivity analysis excluding ASV_4 and *Vibrio* and renormalising confirmed the same pattern: *Tenacibaculum* recovered to baseline by day 3 without exceeding it (Table S5).

Only a single *Tenacibaculum* species, *T. ascidiaceicola* (99.85–100% near-full-length 16S identity), was recovered on non-selective 2216E agar: from pond water on 26–27 June (before mortality), from healthy larvae on 29 June, and from moribund larvae on 2 and 4 July (Table S2). *Tenacibaculum* was never recovered on TCBS: all ten TCBS isolates were *Vibrio*. Even on 2216E agar, this genus grows slowly and is easily overgrown by vibrios. A culture-based survey relying solely on TCBS would therefore recover *Vibrio* abundantly while entirely missing the dominant genus of the rearing water.

### 3.6. The Eyed-Stage Community Converges on a Divergent Rickettsiales-like Lineage

A second unclassified ASV (ASV_3) dominated eyed pediveligers, accounting for 39.3%, 47.1% and 66.9% of sequences in the three tanks (mean 51.1%; CV = 0.28). It was significantly more abundant at the eyed stage than at every earlier stage (Tukey-adjusted *p* = 0.003–0.043).

Its distribution across development was strongly discontinuous. At the healthy D-veliger stage it was detected at 37.0% in one tank and at ≤0.01% in the other two; it was undetectable in all three tanks at both mortality samplings and at early umbo, reappeared at large umbo (21.6%, 4.8%, 0.5%), and dominated all three tanks at the eyed stage (Figure S5). It therefore bloomed after mortality had ceased, rather than during the mortality event itself.

ASV_3 shared no more than 85.6% 16S identity with any described species. Its single best hit in the 16S reference database (*Litoreibacter arenae*, Rhodobacteraceae) exceeded the next, all of them Rickettsiales (*Orientia*, *Anaplasma*, *Ehrlichia*), by less than half a percentage point—a difference without taxonomic meaning at this level of divergence (Table S3). Against the full nucleotide collection, the closest relatives were unambiguously Anaplasmataceae—uncultured clones and *Anaplasma*/*Neoehrlichia* sequences at 86.8–87.6% identity, all uncultured or host-associated (Table S3).

ASV_3 therefore represents a deeply divergent lineage within the Rickettsiales and lies well outside any described genus (Figure 4). A maximum-likelihood phylogeny confirmed this placement: ASV_3 fell within the Rickettsiales, in a clade with *Anaplasma* and *Ehrlichia* supported by 100% bootstrap, and clearly separate from its highest BLAST hit *Litoreibacter* (Rhodobacteraceae) (Figure S6). Its precise position within the order is not resolved, consistent with its deep divergence from all described members. The Anaplasmataceae comprise obligate intracellular symbionts and pathogens of animals, and Rickettsiales-affiliated intracellular associates are widely reported from marine invertebrates [40].

### 3.7. Chlorination of the Feed-Room Supply Removes Tenacibaculum and Is Followed by Dominance of a Sulfur-Oxidising Autotroph

**Figure 5:**
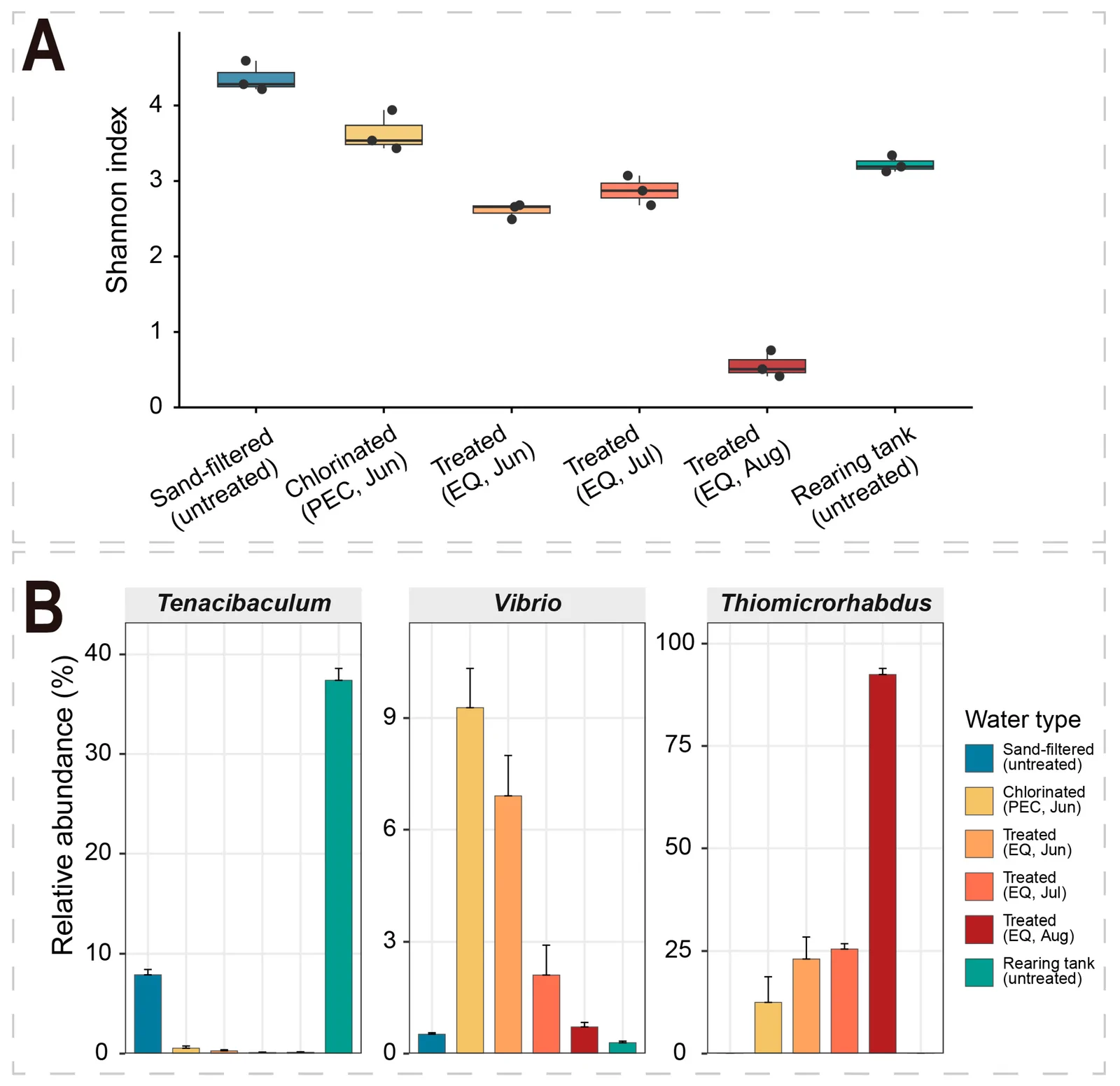
Disinfection selectively restructures the feed-room water community rather than uniformly reducing bacteria. Six water types: untreated sand-filtered water, chlorinated un-neutralised water (PEC, June), neutralised (“treated”, EQ) water at three times (June, July, August), and untreated rearing-tank water. (**A**) Shannon diversity (boxes, median and IQR; points, samples, *n* = 3); the decline in treated water is shown as a trend, not tested (*n* = 3 per group). (**B**) *Tenacibaculum*, *Vibrio* and *Thiomicrorhabdus* (ASV_1) across the same water types (mean + SE, *n* = 3). *Vibrio* and *Thiomicrorhabdus* moved in strict opposition, consistent with compositional replacement within a collapsing community rather than active enrichment of *Vibrio*. Note the different y-axis scales in (**B**). Abundances are relative; absolute quantification would be needed to confirm changes in load.

The microalgae culture facility used the same sand-filtered seawater as the larval rearing tanks; this water was treated with sodium hypochlorite and neutralised with sodium thiosulfate before use. Two sample types were compared with untreated sand-filtered water and with untreated rearing-tank water: water after chlorination but before neutralisation (PEC, June only, *n* = 3), and neutralised water as used (EQ, *n* = 3 per month for three months) (Figure 5).

Diversity declined markedly following treatment. Shannon diversity fell from 4.36 in untreated sand-filtered water to 3.64 in PEC water, and decreased further in EQ water across the season, reaching 0.56 in August (Figure 5A). Observed richness showed a corresponding decline, from 1402 to 1577 ASVs in sand-filtered water to 234–338 in August EQ water (Figure S7). Diversity differed significantly among the six water types (Kruskal–Wallis *p* = 0.006). The 3-versus-3 comparison of sand-filtered against PEC water returned *p* = 0.100, the smallest two-sided value attainable at this sample size (Section 2.7).

The three focal genera responded differently to disinfection (Figure 5B). *Tenacibaculum* fell from 7.9% in untreated sand-filtered water to below 0.6% in all chlorinated (PEC) and treated (EQ) samples, a reduction exceeding 90% that was consistent across all three months. In untreated rearing-tank water drawn from the same sand filter, *Tenacibaculum* reached 37.4%.

*Vibrio* abundance rose in June EQ water (to 6.9%) but declined progressively thereafter, reaching 0.7% by August—a level indistinguishable from that in untreated sand-filtered water. The apparent enrichment of *Vibrio* by chlorination was therefore confined to a single month and was masked when data from all three months were pooled. ASV_1, identified as *Thiomicrorhabdus frisia* (100% 16S identity), was virtually absent from sand-filtered water (0.00%) and from rearing-tank water (0.02%), yet reached 12.5% in PEC water and rose across the season in EQ water, from 23.1% in June to 92.5% in August. Across the entire 78-sample dataset, it exceeded 2000 reads only in EQ and PEC samples.

In EQ water, the relative abundances of *Vibrio* and *Thiomicrorhabdus* moved in strict opposition across the three months: *Vibrio* declined progressively as *Thiomicrorhabdus* increased from approximately a quarter of the community to over 90%. The transient June peak of *Vibrio* is therefore parsimoniously explained as its proportional share of a heavily depleted community, with its subsequent decline reflecting displacement by *Thiomicrorhabdus*; neither trend requires a change in absolute abundance. Determining whether *Vibrio* persists, grows, or merely survives in treated water would require absolute quantification.

The absence of a stored-but-unchlorinated control, however, means that the observed community changes reflect the combined effects of chlorination, neutralisation, and storage, and cannot be attributed to chlorination alone. *Thiomicrorhabdus* is a chemolithoautotrophic sulfur oxidiser, not otherwise noted for chlorine tolerance, and its dominance is at least as consistent with post-treatment succession in stored water (depletion of heterotrophs, oxidation of organic matter, and the emergence of reduced sulfur species) as with selection by chlorine itself.

## 4. Discussion

### 4.1. A Protracted Mortality of Undetermined Cause

Larvae were sampled at the D-veliger stage on 29 June; mortality began the following day and continued until 5 July, with roughly 40% of the cohort lost, after which survivors developed normally through the umbo stages to eyed pediveligers. The two samplings of moribund larvae, conducted on 30 June and 2 July, are therefore best interpreted as successive time points within a single, prolonged mortality event rather than as discrete, independent outbreaks. OsHV-1 was not detected in any of the 18 larval samples, ruling it out as a causative agent and supporting a bacteriological interpretation. A reproducible shift in the larval community accompanied the mortality in all three tanks and reversed once sinking ceased. Within that shift, only one taxon rose as losses accumulated: the undescribed Cryomorphaceae ASV_4, which vanished thereafter. *Vibrio* rose only transiently and had fallen below its healthy-stage abundance two days later, while larvae were still sinking. *Tenacibaculum*, the dominant genus of the rearing water, never exceeded its healthy-stage abundance in larvae at any point. We cannot identify a cause. No change in water temperature, feed, exchange or handling was recorded around onset or cessation; water was exchanged daily throughout. The community data alone cannot distinguish whether this lineage is a primary cause of mortality or merely a mortality-associated opportunist, as proliferation on dying larvae is consistent with both a frank pathogen and a secondary coloniser. The course—losses over several days, spontaneous cessation, and normal development of the remainder—is not that of an acute virulent epizootic, but neither does it exclude a bacterial contribution.

### 4.2. An Undescribed Invertebrate-Associated Lineage Blooms on Moribund Larvae

ASV_4 falls within the Cryomorphaceae, a family of primarily marine Flavobacteriales [41]. No described species exceeds 92.2% 16S identity to it (*Parvicella tangerina*; [42]; 100% query coverage) [34]; the next-closest described relatives—*Croceimicrobium*, *Phaeocystidibacter*, and *Owenweeksia*—fall between 90.2% and 91.0%. Its closest relatives in the wider nucleotide database, however, reach 97–98% and are without exception uncultured environmental clones. Those environmental relatives are, with equal uniformity, epibionts of marine invertebrates: from sea squirts [43], sea urchins, corals, galatheid crabs [44], stalked barnacles [45] and hydrothermal shrimp, spanning four animal phyla to which our data add a fifth, Mollusca. These relatives form a widely distributed, surface-associated group that has escaped cultivation. Its distribution here was host-associated, not environmental: on mortality days ASV_4 was an order of magnitude more abundant in larvae than in the water of the same tanks and was effectively absent from all supply compartments. Proliferation therefore occurred on the host, with low levels in tank water readily explained by release from dying larvae rather than seeding from the water column.

ASV_4 rose at each successive sampling as losses accumulated and had fallen below 1% once sinking ceased—the only taxon to follow the course of the mortality. Two interpretations of this association are possible, and the present data cannot discriminate between them: ASV_4 may actively contribute to the deterioration of larval condition, or it may proliferate opportunistically on larvae that have already become moribund and lost surface integrity. The available evidence favours the latter. On this reading, ASV_4 is a resident host-associated bacterium of *M. gigas* larvae, present at low abundance in healthy animals and held in check under normal physiological conditions. When larvae become moribund, cease swimming and settle to the bottom, surface clearance fails and it overgrows, reaching more than half of the larval community before disappearing completely once mortality ends. This is characteristic of a pathobiont released from host control rather than of an invading pathogen. A comparable sequence has been characterised in situ during Pacific oyster mortality syndrome, in which an initial insult is followed by opportunistic colonisation of compromised hosts [46]; that syndrome affects juveniles and is initiated by OsHV-1, which was absent from our samples. The mechanism we propose does not require ASV_4 to be a pathogen. ASV_4’s proliferation may therefore mark the loss of host surface clearance rather than cause it—and if so, it would constitute a sensitive indicator of larval condition rather than an agent of disease. Nothing in the ecology of its closest relatives, which are commensal or symbiotic epibionts in the systems where they have been studied, predicts virulence.

Three approaches would distinguish these possibilities. Scanning electron microscopy or fluorescence in situ hybridisation of moribund larvae would establish whether a bacterial mat is present on the larval surface and requires no more than fixed material. Isolation would permit challenge experiments, though no member of this lineage has been cultured and it will require oligotrophic marine media. Absolute quantification by qPCR would separate proliferation from compositional displacement. A caveat: larvae were rinsed twice in sterile seawater, which removes loosely adherent particles but not firmly attached epibionts; whole-larva DNA extraction cannot distinguish surface-colonising from tissue-associated bacteria. This limitation applies particularly to ASV_4, whose closest relatives are epibionts; we do not claim that ASV_4 invaded larval tissue.

### 4.3. Vibrio: A Plausible but Unproven Contributor at the Onset of Mortality

*Vibrio* is the most extensively documented bacterial agent of larval mortality in bivalve hatcheries; several species reproducibly kill *M. gigas* larvae in challenge experiments, and vibriosis-linked epizootics have been described for decades [2,3,8,10,11,12]. In this context, the rise in *Vibrio* abundance across all three tanks at the onset of mortality cannot be easily dismissed, and *Vibrio* must be considered a plausible contributor to the event. Although the rise was directionally consistent across replicates, it did not survive multiple-test correction (Table S5). The consistency among replicate tanks is arguably more informative than any single *p*-value, yet neither observation establishes causation.

The same organisms occur in healthy larvae. *Vibrio* was isolated from moribund larvae at both mortality samplings—*V. alginolyticus* on day 1 and *V. chagasii* on day 3—as well as from healthy larvae and from pond water (Table S2). Isolation from dying animals establishes viability and host association; it does not distinguish a pathogen from a commensal. This limitation is general to culture-based association studies and is not always made explicit.

*V. alginolyticus*, *V. cyclitrophicus*, *V. mytili*, *V. toranzoniae*, *V. ulleungensis* and *V. chagasii* are widespread in coastal seawater and in healthy bivalves [47]. None belongs to the small group—*V. coralliilyticus*, *V. tubiashii*, *V. bivalvicida*—for which high virulence towards bivalve larvae is established. That group remains the one most consistently recovered from acute larval mortality in *M. gigas* hatcheries, as exemplified by a recent epizootic in Japan, where *V. coralliilyticus* isolates proved highly virulent in challenge tests [48]. Their presence is consistent with opportunistic proliferation in compromised hosts rather than with invasion by a virulent strain [49], although virulence cannot be inferred from 16S identity. The bloom did not persist through the mortality. *Vibrio* was elevated on the first day of mortality and had fallen below its healthy-stage abundance by the third, while larvae continued to die and cumulative losses continued to rise. A taxon driving mortality would not be expected to subside while mortality proceeds. Its transient rise is more readily interpreted as an early, self-limiting bloom—whether contributory, coincidental, or a consequence of the initial deterioration in host condition—than as the driver of the mortality.

*Vibrio* also remained below 3.1% in every water compartment, offering no evident route by which a bloom might have been seeded from the supply; broodstock pond water, at 3.02%, was the richest source sampled and—together with the recovery of multiple *Vibrio* species from broodstock tissue—is the most plausible point of entry [50].

### 4.4. The Dominant Genus of the Rearing Water Is One That Culture Would Not Have Identified

Larval and water samples were plated in parallel on non-selective marine agar (2216E) and Vibrio-selective TCBS agar. *Tenacibaculum* was recovered repeatedly on 2216E from pond water, microalgae culture tanks, and both healthy and moribund larvae, but never on TCBS, which yielded only *Vibrio* (Table S2). This outcome illustrates the bias inherent in Vibrio-selective surveys: every one of the ten TCBS isolates in this study was a *Vibrio*, and not a single *Tenacibaculum* was recovered on that medium, although it was readily cultured from the same samples on non-selective agar (Table S2). A survey relying on TCBS would therefore report abundant *Vibrio* while entirely missing the most abundant genus in the rearing water. Surveillance strategies that rely on selective culture cannot, in principle, detect the taxa they select against, and the bivalve hatchery microbiology literature—which has focused predominantly on *Vibrio*—has been shaped in part by this constraint.

Despite its dominance in the rearing water, *Tenacibaculum* is not implicated in the mortality. In larvae, *Tenacibaculum* never exceeded its healthy-stage abundance, and its apparent dip during mortality can be explained by compositional displacement as ASV_4 and *Vibrio* bloomed. In tank water, abundance was highest when larvae were healthy and declined thereafter, so its abundance does not track mortality. *Tenacibaculum maritimum* is a well-characterised pathogen of marine fish, causing erosive lesions through gliding motility, surface adhesion, and extracellular enzymes [14]. The species isolated here, *T. ascidiaceicola*, was originally described from an ascidian, the golden sea squirt *Halocynthia aurantium* [51], and has no known pathogenicity in any host. It was recovered from the broodstock pond and microalgae culture tanks before mortality began, and from both healthy and moribund larvae, a distribution consistent with an established resident of the system. High environmental abundance, membership in a genus that contains pathogens, and recovery from moribund animals do not, by themselves, constitute evidence of pathogenicity; no pathogenic role is inferred here.

### 4.5. The Eyed Stage Is Dominated by a Divergent Rickettsiales-like Lineage

By the eyed pediveliger stage, a single ASV (ASV_3) accounted for 39.3–66.9% of larval sequences in all three tanks. It shares no more than 85.6% identity with any described species, and its closest environmental relatives are unambiguously Anaplasmataceae; it therefore falls well outside any described genus (Figure 4, Table S3).

A maximum-likelihood phylogeny placed ASV_3 within the Rickettsiales, in a clade with *Anaplasma* and *Ehrlichia* supported by 100% bootstrap, and clearly separate from its highest BLAST hit *Litoreibacter* (Rhodobacteraceae) (Figure S6). This confirms the Rickettsiales-like assignment; its precise position within the order is not resolved, consistent with its deep divergence from all described members.

The Anaplasmataceae comprise obligate intracellular symbionts and pathogens of animals, and Rickettsiales-affiliated intracellular associates are widely reported from marine invertebrates [40]. The temporal pattern of ASV_3 is consistent with such a lifestyle: undetectable in all three tanks at both mortality samplings, reappearing at the large umbo stage, and dominating the eyed stage. This suggests a stage-specific association with healthy, post-crisis larvae rather than with disease. Its near-absence from two of three tanks at the D-veliger stage, and presence at 37% in the third, suggests that colonisation is stochastic in timing.

### 4.6. Chlorination Removes One Candidate and Not the Other, and Remakes the Community

The microalgae culture facility drew from the same sand-filtered supply as the larval tanks and subjected it to chlorination, thereby creating an unplanned contrast between treated and untreated water from a common source.

Under this treatment, *Tenacibaculum* was reduced by more than 90% across all three months, whereas it remained the dominant genus in the untreated rearing tanks fed by the same sand-filtered supply. Should *Tenacibaculum* ever be shown to pose a threat, this reservoir would therefore be chemically tractable.

In contrast, *Vibrio* was not controlled by chlorination. Its relative abundance rose in June but declined progressively, falling to levels indistinguishable from untreated sand-filtered water by August. Over the same period, *Thiomicrorhabdus* increased to dominance, and its abundance moved in strict opposition to that of *Vibrio*. The transient June peak of *Vibrio* is therefore most parsimoniously interpreted as its proportional share of a heavily depleted community rather than as absolute growth [52]; when data from all three months are pooled, this temporal pattern is entirely obscured.

Disinfection thus did more than remove taxa; it created a new dominant. *Thiomicrorhabdus frisia* was absent from untreated water and from the rearing tanks, yet came to dominate the feed-room supply by August (>90% of sequences), at which point Shannon diversity had collapsed. A diverse heterotrophic assemblage had been replaced by a near-monoculture of a chemolithoautotrophic sulfur oxidiser.

Attributing these changes solely to chlorine, however, would be premature. EQ water was subject to a combined sequence of chlorination, neutralisation with thiosulfate, and storage before use, and no stored-but-unchlorinated control was included. *Thiomicrorhabdus* is not a taxon noted for chlorine tolerance; it is an autotroph that oxidises reduced sulfur compounds [53]. Its dominance is at least as consistent with succession in stored, heterotroph-depleted water (in which thiosulfate itself is a reduced sulfur species and a plausible substrate) as with selection by the oxidant. Distinguishing these alternatives would require a control not included in the present study, and the practice of neutralising with thiosulfate warrants examination in its own right.

These findings reinforce the principle that disinfection is not a uniform reduction in bacterial load but a selective process whose outcome depends on which taxa tolerate it and what proliferates afterwards [54,55]; the residual community may be less diverse and functionally quite different from the original assemblage. Furthermore, in a community whose total biomass has collapsed, relative abundance data alone will make survivors appear to be winners.

### 4.7. Limitations

Without challenge experiments, none of the organisms identified here can be designated a pathogen; any such assignments remain provisional, and the isolates we obtained provide the material needed for confirmatory testing. Virulence-associated phenotypes, including extracellular protease and metalloprotease activity, were likewise not assayed; phenotypic screening combined with challenge and absolute quantification is the logical next step. Several caveats arise from the study design. The study followed a single cohort in one hatchery over a single season, and broader generalisation is not intended. The absence of a healthy control group means that mortality-specific changes cannot be formally distinguished from those linked to ontogeny, though repeated sampling of the same three tanks before and after the event provides a partial internal baseline. Within this baseline, ASV_4 was confined to the mortality interval and did not reappear at any other developmental transition; a bloom coinciding with the normal D-veliger to umbo transition cannot be excluded. Additionally, sequence-derived abundances are compositional, so claims of absolute proliferation require qPCR confirmation, and with three tanks several contrasts cannot reach significance regardless of effect size; we have accordingly avoided causal language. Finally, the temporal resolution is incomplete: sinking continued for three days after the last moribund sample was taken, so the final phase of the mortality event was not observed.

## 5. Conclusions

Larval mortality in bivalve hatcheries is routinely attributed to *Vibrio* and monitored by selective culture. Across a full larval rearing cycle of *M. gigas* at a commercial hatchery, neither assumption held. *Vibrio* rose only briefly at the onset of a protracted mortality event, then fell below its healthy-stage abundance while larvae were still dying. That trajectory is more consistent with a resident member of the larval microbiota than with the agent driving the die-off. *Tenacibaculum* dominated the rearing water but showed no association with mortality, was systematically under-recovered on Vibrio-selective medium, and was the taxon that chlorination removed while leaving *Vibrio* uncontrolled. The only taxon to rise above the healthy baseline as mortality proceeded was an undescribed, uncultured Cryomorphaceae lineage (ASV_4), invisible to culture-based monitoring. These are associations from a single mortality event at one facility, and ASV_4 remains a candidate rather than a demonstrated pathogen. They nonetheless show that a Vibrio-centred, culture-based paradigm can monitor a mortality event and guide water-supply disinfection practices without ever seeing the taxon that tracked it. Whole-community, culture-independent surveillance is therefore needed to establish which organisms in bivalve hatcheries are worth culturing and worth targeting.

## Data Availability

The raw 16S rRNA gene amplicon sequencing data generated in this study have been deposited in the NCBI Sequence Read Archive (SRA) under BioProject accession PRJNA1496542.

## Supplementary Materials

The following supporting information can be downloaded at: https://www.mdpi.com/article/10.3390/pathogens15080830/s1. Supplementary Methods; Figure S1: Rarefaction curves of observed ASV richness by sample type; Figure S2: Family-level community composition across the hatchery system; Figure S3: Family-level composition of rearing-tank water across larval development; Figure S4: *Tenacibaculum* and *Vibrio* abundance along the water-supply chain at three sampling times; Figure S5: ASV_4 and ASV_3 abundance across larval development; Figure S6: Maximum-likelihood phylogeny of ASV_3 and ASV_4; Figure S7: Residual community in disinfected feed-room water (EQ) across three sampling times, compared with untreated sand-filtered water; Table S1: Sample metadata for the 78 samples; Table S2: Bacterial isolates and near-full-length 16S rRNA gene identification; Table S3: BLAST-based identification of the dominant undescribed larval ASVs; Table S4: ASVs removed as putative contaminants; Table S5: Summary of statistical tests.
