# Supplementary material for: Bacterial Communities Across the Production Chain of a Pacific Oyster (Magallana gigas) Hatchery During a Larval Mortality Event
Source: Pathogens. 2026 Aug 7;15(8):830. doi: 10.3390/pathogens15080830 (PMC13516132; doi:10.3390/pathogens15080830)
Supplement: Supplementary file 1 [file pathogens-15-00830-s001.zip › Supplementary_Figure.pdf]

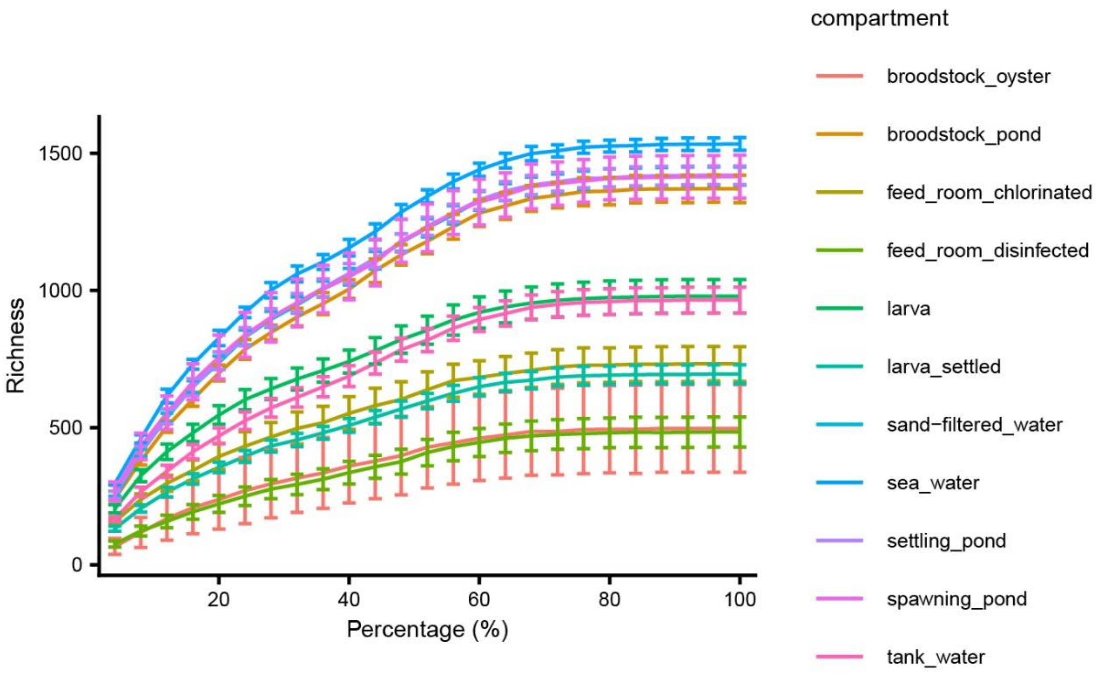

Figure S1. Rarefaction curves of observed ASV richness for all samples, grouped by sample type. Curves approaching a plateau indicate that the sequencing depth (rarefied to 40,000 reads per sample) was sufficient to capture the bacterial diversity of each sample. Each line represents a sample; colours denote sample type.

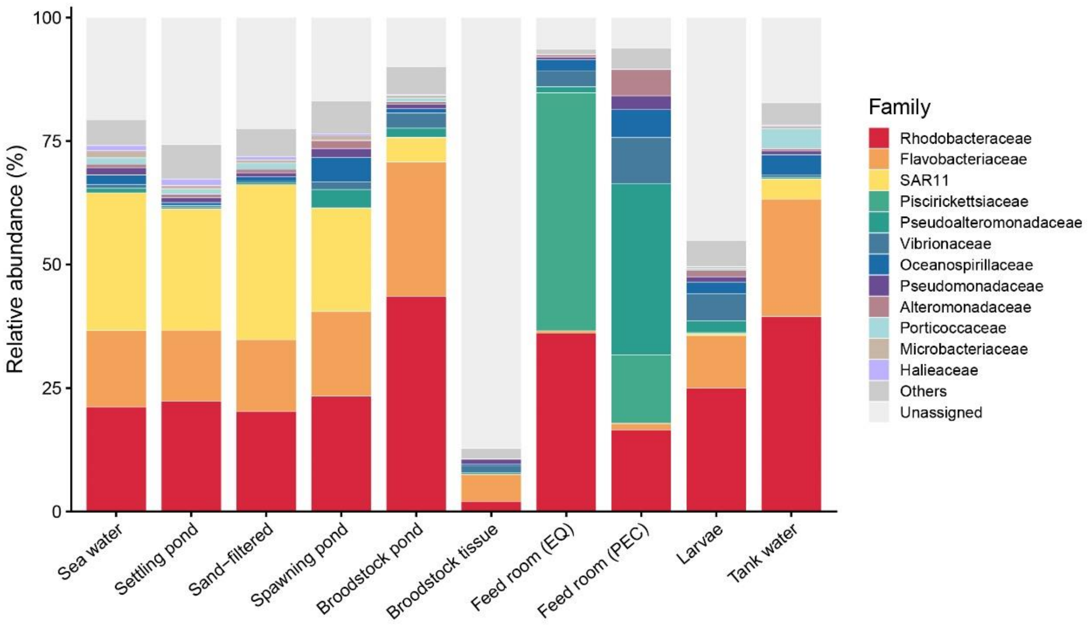

Figure S2. Overview of the bacterial community composition (family level) across the entire hatchery system. Bars represent group means of relative abundance for ten sample categories, ordered along the system flow: sea water → settling pond → sand-filtered water → spawning pond → broodstock pond → broodstock tissue → feed-room disinfected water (EQ) → feed-room chlorinated water (PEC) → larvae → tank water. The twelve most abundant families are shown; remaining families are grouped as "Others", and sequences that could not be classified to family are shown as "Unassigned".

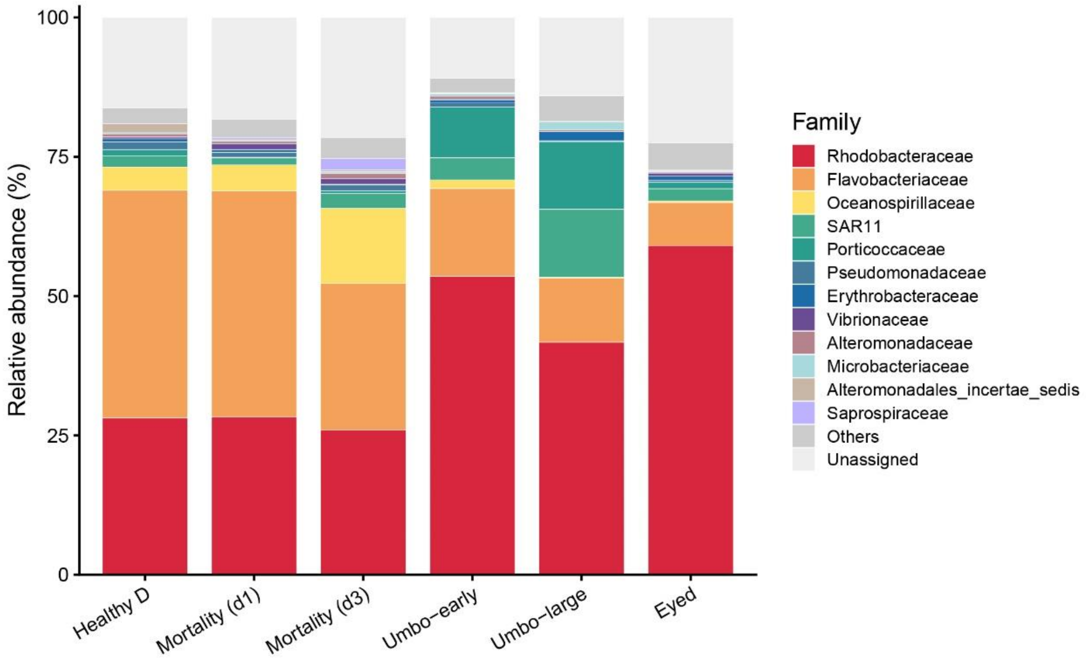

Figure S3. Bacterial community composition (family level) of rearing-tank water across the C-group larval developmental stages (healthy D-veliger, mortality day 1, mortality day 3, early umbo, large umbo, eyed). This water-phase counterpart to the larval community (Figure 2A) shows the environmental microbial context in which the larvae developed. Bars represent stage means (*n* = 3). The two mortality time points (day 1, 30 June; day 3, 2 July) are two samplings within a single protracted mortality event.

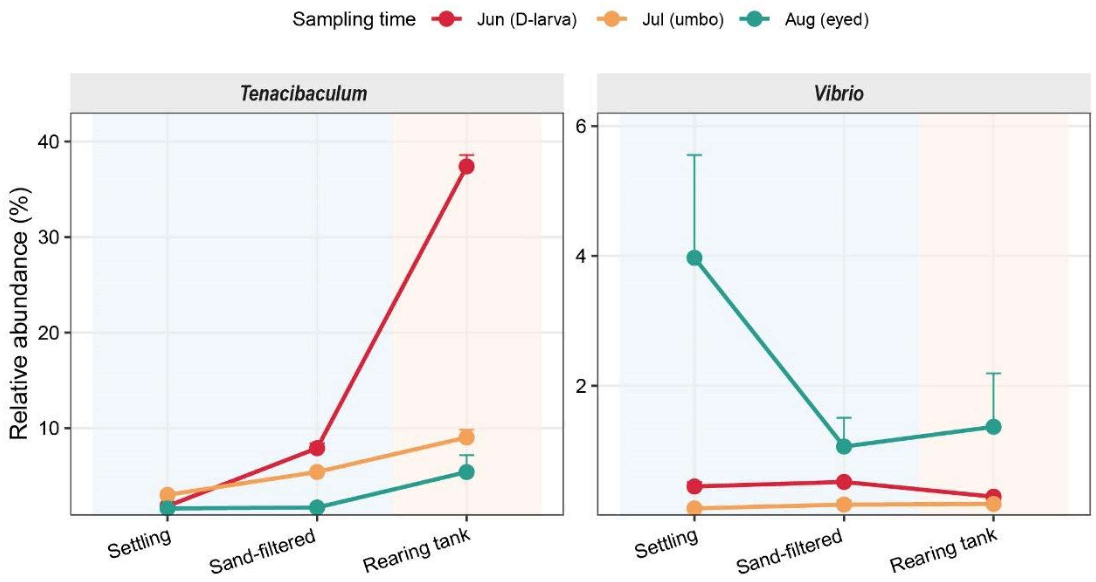

Figure S4. Relative abundance of the two candidate opportunistic pathogens (*Tenacibaculum* and *Vibrio*, each summed across all assigned ASVs) along the water-supply chain (settling pond → sand filtration → rearing tank) at three sampling times (June, July, August). Points and lines show means ± SE ($n = 3$). *Tenacibaculum* remained low throughout the incoming supply and reached its highest abundance in rearing-tank water. Note the different y-axis scales for the two taxa. Abundances are relative; absolute quantification (e.g., qPCR) would be required to confirm these patterns.

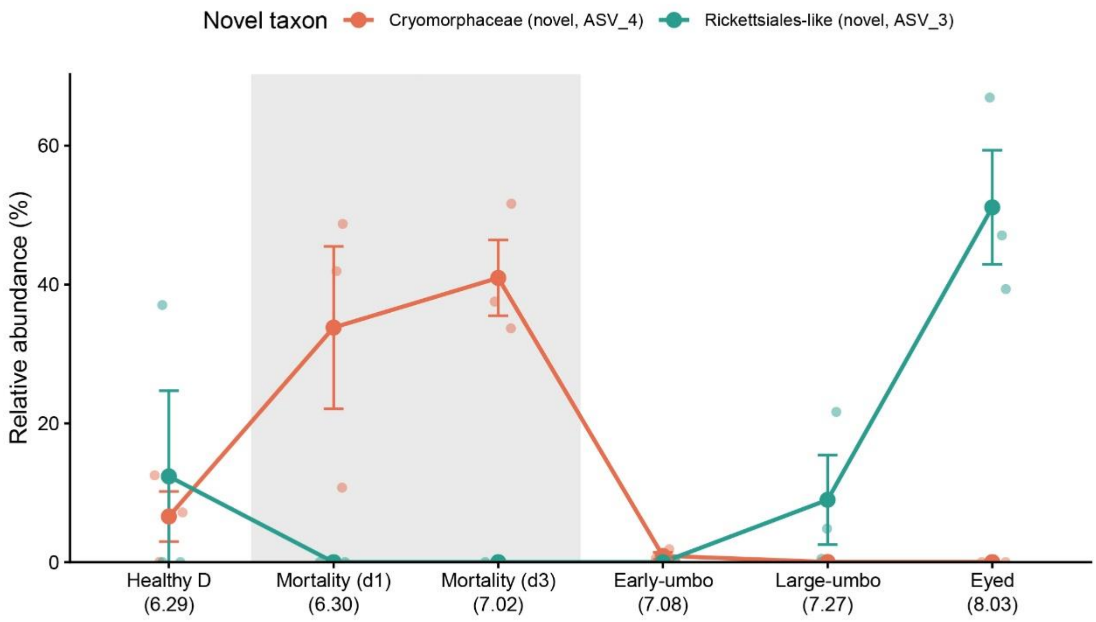

Figure S5. Relative abundance of two novel dominant taxa across C-group larval development: the novel Cryomorphaceae (ASV_4) and the novel Rickettsiales-like taxon (ASV_3). Lines connect stage means; individual points show the three biological replicates. Grey bands mark the two mortality samplings (settled moribund larvae, 30 June and 2 July), representing two time points within a single protracted mortality event. The novel Cryomorphaceae rose as mortality accumulated (up to ~50%) and was near-absent at normal developmental stages, whereas the Rickettsiales-like taxon appeared sporadically and heterogeneously in early larvae before dominating at the eyed stage (~50%). Neither taxon could be assigned to a described genus (Table S3). Abundances are relative (16S V4–V5, rarefied to 40,000 reads per sample); *n* = 3.

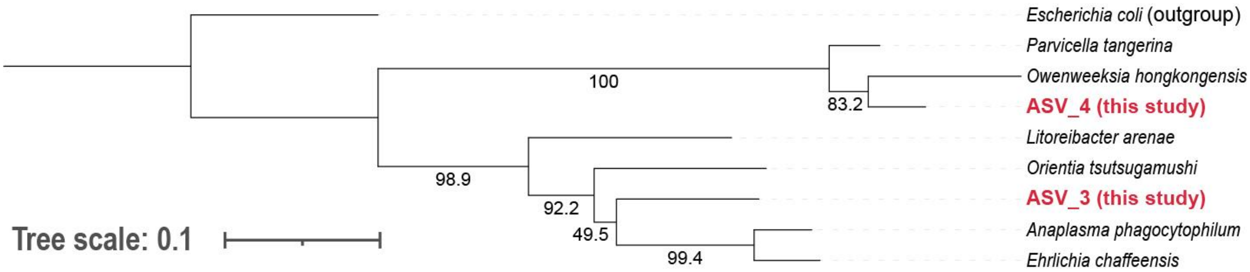

Figure S6. Maximum-likelihood phylogeny of ASV_3 and ASV_4 based on 16S rRNA gene sequences, with representative Rickettsiales and Cryomorphaceae reference taxa and *Escherichia coli* as the outgroup. The tree was inferred with IQ-TREE under the TIM3+F+G4 model; node values are ultrafast bootstrap support (%). ASV_3 (this study) falls within the Rickettsiales, in a well-supported clade with *Anaplasma* and *Ehrlichia*, and separate from its highest BLAST hit *Litoreibacter arenae* (Rhodobacteraceae), confirming its Rickettsiales-like assignment; its precise position within the order is not resolved. ASV_4 (this study) falls within the Cryomorphaceae. Scale bar, substitutions per site.

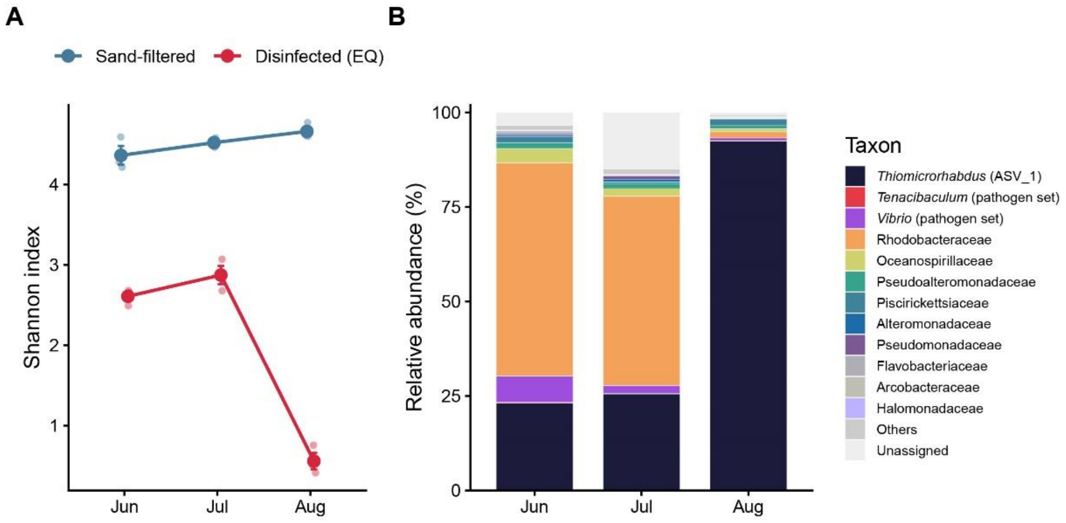

Figure S7. Residual bacterial community in feed-room disinfected water (EQ) across three sampling times, compared with untreated sand-filtered water. (A) Shannon diversity of EQ versus sand-filtered water at each time point (mean ± SE, $n = 3$); chlorination markedly and progressively reduced diversity. (B) Community composition of EQ water (relative abundance), with the two candidate pathogens (*Vibrio*, *Tenacibaculum*; each summed across their assigned ASVs) and the chlorine-tolerant chemolithoautotroph *Thiomicrorhabdus* (ASV_1) shown separately, remaining taxa at family level. The community became progressively simplified, culminating in near-complete dominance by *Thiomicrorhabdus* (~92%) in August, illustrating that disinfection selects for specific tolerant taxa rather than uniformly reducing all bacteria.
