# Supplementary material for: Bacterial Communities Across the Production Chain of a Pacific Oyster (Magallana gigas) Hatchery During a Larval Mortality Event
Source: Pathogens. 2026 Aug 7;15(8):830. doi: 10.3390/pathogens15080830 (PMC13516132; doi:10.3390/pathogens15080830)
Supplement: Supplementary file 1 [file pathogens-15-00830-s001.zip › Supplementary_Methods.pdf]

Supplementary Methods

Contaminant removal: The removed genera comprised (i) reagent/kitome taxa (*Ralstonia*, *Bradyrhizobium*, *Methylobacterium*, *Sphingomonas*, *Brevundimonas*, *Stenotrophomonas*, *Delftia*, among others; full list in Table S4); (ii) human-associated taxa (*Cutibacterium*, *Staphylococcus*, *Streptococcus*, *Corynebacterium*, *Micrococcus*); and (iii) freshwater/soil Comamonadaceae and relatives (*Hydrogenophaga*, *Acidovorax*, *Comamonas*, *Curvibacter*, *Limnohabitans*). A minor subset (0.18% of reads) matched contaminant genera only at low confidence (< 0.5). The low-threshold annotation was used for this screen because the conservative (0.8) annotation collapses low-confidence genus assignments to higher ranks, leaving many contaminant ASVs unidentified.

Gut-associated taxa: A further 63 ASVs were assigned to mammalian-gut anaerobes (*Alistipes*, Lachnospiraceae). These ASVs (SINTAX confidence ⩾ 0.9 for 60 of them) do not appear in the reference contaminant list [33]. They accounted for 8,600 reads in total (0.276% of all sequences; maximum 4.5% in any single sample) and were distributed sporadically across inflow water, broodstock tissue and larvae of several stages, with no association with any developmental stage or compartment. They were judged to reflect low-level cross-contamination during multiplexed sequencing and were retained; their exclusion alters no result reported here.
