# Supplementary material for: Bacterial Communities Across the Production Chain of a Pacific Oyster (Magallana gigas) Hatchery During a Larval Mortality Event
Source: Pathogens. 2026 Aug 7;15(8):830. doi: 10.3390/pathogens15080830 (PMC13516132; doi:10.3390/pathogens15080830)
Supplement: Supplementary file 1 [file pathogens-15-00830-s001.zip › Supplementary_Table.pdf]

Table S1. Sample metadata for the 78 samples analysed in this study

sample_name	date	stage	sample_type	compartment	analysis_block	diseased
pbHQ1	25 June	pre-breeding	water	sea_water	watersource	
pbHQ2	25 June	pre-breeding	water	sea_water	watersource	
pbHQ3	25 June	pre-breeding	water	sea_water	watersource	
pbLQ1	25 June	pre-breeding	water	settling_pond	watersource	
pbLQ2	25 June	pre-breeding	water	settling_pond	watersource	
pbLQ3	25 June	pre-breeding	water	settling_pond	watersource	
pbLH1	25 June	pre-breeding	water	sand-filtered_water	watersource	
pbLH2	25 June	pre-breeding	water	sand-filtered_water	watersource	
pbLH3	25 June	pre-breeding	water	sand-filtered_water	watersource	
pbEQ1	25 June	pre-breeding	water	feed_room_disinfected	watersource	
pbEQ2	25 June	pre-breeding	water	feed_room_disinfected	watersource	
pbEQ3	25 June	pre-breeding	water	feed_room_disinfected	watersource	
pbPEC1	25 June	pre-breeding	water	feed_room_chlorinated	watersource	
pbPEC2	25 June	pre-breeding	water	feed_room_chlorinated	watersource	
pbPEC3	25 June	pre-breeding	water	feed_room_chlorinated	watersource	
pbCW1	26 June	Broodstock	water	broodstock_pond	origin	
pbCW2	26 June	Broodstock	water	broodstock_pond	origin	
pbCW3	26 June	Broodstock	water	broodstock_pond	origin	
pbCL1	26 June	Broodstock	water	spawning_pond	origin	
pbCL2	26 June	Broodstock	water	spawning_pond	origin	
pbCL3	26 June	Broodstock	water	spawning_pond	origin	
pbCP1	26 June	Broodstock	tissue	broodstock_oyster	origin	
pbCP2	26 June	Broodstock	tissue	broodstock_oyster	origin	
pbCP3	26 June	Broodstock	tissue	broodstock_oyster	origin	
dsCC1	29 June	D-veliger	tissue	larva	core_629	no
dsCC2	29 June	D-veliger	tissue	larva	core_629	no
dsCC3	29 June	D-veliger	tissue	larva	core_629	no
dsCFC1	29 June	D-veliger	water	tank_water	core_629	no
dsCFC2	29 June	D-veliger	water	tank_water	core_629	no
dsCFC3	29 June	D-veliger	water	tank_water	core_629	no
ds630CLCL1	30 June	D-veliger	tissue	larva_settled	disease	diseased_settled
ds630CLCL2	30 June	D-veliger	tissue	larva_settled	disease	diseased_settled
ds630CLCL3	30 June	D-veliger	tissue	larva_settled	disease	diseased_settled
ds630CFCL1	30 June	D-veliger	water	tank_water	disease	diseased_tank
ds630CFCL2	30 June	D-veliger	water	tank_water	disease	diseased_tank
ds630CFCL3	30 June	D-veliger	water	tank_water	disease	diseased_tank
ds702CLCL1	02 July	D-veliger	tissue	larva_settled	disease	diseased_settled
ds702CLCL2	02 July	D-veliger	tissue	larva_settled	disease	diseased_settled
ds702CLCL3	02 July	D-veliger	tissue	larva_settled	disease	diseased_settled
ds702CFCL1	02 July	D-veliger	water	tank_water	disease	diseased_tank
ds702CFCL2	02 July	D-veliger	water	tank_water	disease	diseased_tank

ds702CFCL3	02 July	D-veliger	water	tank_water	disease	diseased_tank
euLQ1	07 July	Early_umbo	water	settling_pond	watersource	
euLQ2	07 July	Early_umbo	water	settling_pond	watersource	
euLQ3	07 July	Early_umbo	water	settling_pond	watersource	
euLH1	07 July	Early_umbo	water	sand-filtered_water	watersource	
euLH2	07 July	Early_umbo	water	sand-filtered_water	watersource	
euLH3	07 July	Early_umbo	water	sand-filtered_water	watersource	
euEQ1	07 July	Early_umbo	water	feed_room_disinfected	watersource	
euEQ2	07 July	Early_umbo	water	feed_room_disinfected	watersource	
euEQ3	07 July	Early_umbo	water	feed_room_disinfected	watersource	
euCC1	08 July	Early_umbo	tissue	larva	core_708	no
euCC2	08 July	Early_umbo	tissue	larva	core_708	no
euCC3	08 July	Early_umbo	tissue	larva	core_708	no
euCFC1	08 July	Early_umbo	water	tank_water	core_708	no
euCFC2	08 July	Early_umbo	water	tank_water	core_708	no
euCFC3	08 July	Early_umbo	water	tank_water	core_708	no
luCC1	27 July	Large_umbo	tissue	larva	core_727	no
luCC2	27 July	Large_umbo	tissue	larva	core_727	no
luCC3	27 July	Large_umbo	tissue	larva	core_727	no
luCFC1	27 July	Large_umbo	water	tank_water	core_727	no
luCFC2	27 July	Large_umbo	water	tank_water	core_727	no
luCFC3	27 July	Large_umbo	water	tank_water	core_727	no
epLQ1	03 Aug	Eyed_pedoveliger	water	settling_pond	watersource	
epLQ2	03 Aug	Eyed_pedoveliger	water	settling_pond	watersource	
epLQ3	03 Aug	Eyed_pedoveliger	water	settling_pond	watersource	
epLH1	03 Aug	Eyed_pedoveliger	water	sand-filtered_water	watersource	
epLH2	03 Aug	Eyed_pedoveliger	water	sand-filtered_water	watersource	
epLH3	03 Aug	Eyed_pedoveliger	water	sand-filtered_water	watersource	
epEQ1	03 Aug	Eyed_pedoveliger	water	feed_room_disinfected	watersource	
epEQ2	03 Aug	Eyed_pedoveliger	water	feed_room_disinfected	watersource	
epEQ3	03 Aug	Eyed_pedoveliger	water	feed_room_disinfected	watersource	
epCC1	03 Aug	Eyed_pedoveliger	tissue	larva	core_803	no
epCC2	03 Aug	Eyed_pedoveliger	tissue	larva	core_803	no
epCC3	03 Aug	Eyed_pedoveliger	tissue	larva	core_803	no
epCFC1	03 Aug	Eyed_pedoveliger	water	tank_water	core_803	no
epCFC2	03 Aug	Eyed_pedoveliger	water	tank_water	core_803	no
epCFC3	03 Aug	Eyed_pedoveliger	water	tank_water	core_803	no

Table S2. Bacterial isolates recovered from water, broodstock and larvae, identified by near-full-length 16S rRNA gene sequencing.

Isolate	Source	Condition	Date	Medium	Best 16S match
Mg-1	Sea_Water	—	25-June	2216E	*Vibrio ulleungensis* strain 188UL20-2
Mg-2	Sea_Water	—	25-June	2216E	*Tenacibaculum lutimaris* strain TF-26
Mg-3	Sea_Water	—	25-June	2216E	*Phaeobacter piscinae* strain 27-4
Mg-4	Sea_Water	—	25-June	2216E	*Vibrio alginolyticus* strain NBRC 15630
Mg-5	Sea_Water	—	25-June	TCBS	*Vibrio alginolyticus* strain NBRC 15630
Mg-6	Settling_pond	—	25-June	2216E	*Vibrio alginolyticus* strain NBRC 15630
Mg-7	Settling_pond	—	25-June	2216E	*Pseudoalteromonas tetraodonis* GFC strain KMM 458
Mg-8	Settling_pond	—	25-June	2216E	*Bacillus aequororis* strain M-8
Mg-9	Settling_pond	—	25-June	2216E	*Streptococcus infantis* ATCC 700779 strain CCUG 39817
Mg-10	Settling_pond	—	25-June	2216E	*Alteromonas stellipolaris* strain R10SW13
Mg-11	Settling_pond	—	25-June	TCBS	*Vibrio cyclitrophicus* strain LMG 21359
Mg-12	Settling_pond	—	25-June	TCBS	*Vibrio alginolyticus* strain ATCC 17749
Mg-13	Sand-filtered_water	—	25-June	2216E	*Mesobacillus thioparans* strain BMP-1
Mg-14	Sand-filtered_water	—	25-June	2216E	*Polaribacter dokdonensis* DSW-5
Mg-15	Broodstock_pond	—	26-June	2216E	*Vibrio mytili* strain CECT 632
Mg-16	Broodstock_pond	—	26-June	2216E	*Vibrio cyclitrophicus* strain LMG 21359
Mg-17	Broodstock_pond	—	26-June	2216E	*Tenacibaculum ascidiaceicola* strain RSS1-6
Mg-18	Broodstock_pond	—	26-June	2216E	*Vibrio alginolyticus* strain NBRC 15630
Mg-19	Broodstock_pond	—	26-June	2216E	*Vibrio cyclitrophicus* strain LMG 21359
Mg-20	Broodstock_pond	—	26-June	2216E	*Vibrio alginolyticus* strain NBRC 15630
Mg-21	Broodstock_pond	—	26-June	2216E	*Tenacibaculum ascidiaceicola* strain RSS1-6
Mg-22	Broodstock_pond	—	26-June	TCBS	*Vibrio alginolyticus* strain NBRC 15630
Mg-23	Broodstock_pond	—	26-June	TCBS	*Vibrio cyclitrophicus* strain LMG 21359
Mg-24	Broodstock	—	26-June	2216E	*Vibrio mytili* strain CECT 632
Mg-25	Broodstock	—	26-June	2216E	*Pseudoalteromonas* marina strain mano4
Mg-26	Broodstock	—	26-June	TCBS	*Vibrio toranzoniae* strain Vb 10.8
Mg-27	Feed_room_chlorinated	—	26-June	2216E	*Vibrio alginolyticus* strain NBRC 15630
Mg-28	Feed_room_chlorinated	—	26-June	2216E	*Pseudoalteromonas* marina strain mano4
Mg-29	Feed_room_chlorinated	—	26-June	TCBS	*Vibrio alginolyticus* strain NBRC 15630
Mg-30	feed_room_disinfected	—	26-June	2216E	*Pseudoalteromonas tetraodonis* GFC strain KMM 458
Mg-31	feed_room_disinfected	—	26-June	2216E	*Polaribacter marinaquae* strain RZW3-2
Mg-32	feed_room_disinfected	—	26-June	2216E	*Alteromonas abrolhosensis* strain PEL67E
Mg-33	feed_room_disinfected	—	26-June	2216E	*Tenacibaculum halocynthiae* strain P-

					R2A1-2
Mg-34	microalgae culture tank	—	27-June	2216E	*Tenacibaculum ascidiaceicola* strain RSS1-6
Mg-35	microalgae culture tank	—	27-June	2216E	*Tenacibaculum ascidiaceicola* strain RSS1-6
Mg-36	Larvae	no mortality	29-June	2216E	*Tenacibaculum ascidiaceicola* strain RSS1-6
Mg-37	Larvae	no mortality	29-June	2216E	*Pseudoalteromonas shioyasakiensis* strain SE3
Mg-38	Larvae	no mortality	29-June	2216E	*Alteromonas abrolhosensis* strain PEL67E
Mg-39	Larvae	no mortality	29-June	2216E	*Vibrio alginolyticus* strain NBRC 15630
Mg-40	Larvae	no mortality	29-June	2216E	*Alteromonas tagae* strain BCRC 17571
Mg-41	Larvae	no mortality	29-June	2216E	*Pseudoalteromonas piratica* strain OCN003
Mg-42	Larvae	no mortality	29-June	TCBS	*Vibrio alginolyticus* strain NBRC 15630
Mg-43	Larvae	mortality d1	30-June	2216E	*Pseudophaeobacter arcticu*s DSM 23566 strain 20188
Mg-44	Larvae	mortality d1	30-June	2216E	*Pseudophaeobacter leonis* strain 306
Mg-45	Larvae	mortality d1	30-June	TCBS	*Vibrio alginolyticus* strain NBRC 15630
Mg-46	Larvae	mortality d3	2-July	2216E	*Tenacibaculum ascidiaceicola* strain RSS1-6
Mg-47	Larvae	mortality d3	2-July	2216E	*Vibrio chagasii* strain LMG 21353
Mg-48	Larvae	mortality d3	2-July	2216E	*Tenacibaculum ascidiaceicola* strain RSS1-6
Mg-49	Larvae	mortality d3	2-July	2216E	*Tenacibaculum ascidiaceicola* strain RSS1-6
Mg-50	Larvae	mortality d3	2-July	2216E	*Tenacibaculum ascidiaceicola* strain RSS1-6
Mg-51	Larvae	mortality d3	2-July	TCBS	*Vibrio chagasii* strain LMG 21353

Condition refers to larval samples only: Healthy (29 June) or Moribund (mortality day 1, 30 June; day 3, 2 July); "—" indicates non-larval samples. Medium: 2216E, non-selective marine agar; TCBS, Vibrio-selective agar. Isolates were identified to the closest species by near-full-length 16S rRNA gene sequencing.

Table S3. BLAST-based identification of the dominant undescribed larval ASVs.

ASV	Taxonomic assignment	Closest described species (accession)	Ident (%)	Cover (%)	Closest environmental clone (accession; source)	Ident (%)	Cover (%)
ASV_1	*Thiomicrorhabdus* (reference)	*Thiomicrorhabdus frisia* (NR_028679)	100.0	100	Uncultured clone (e.g. AY216451)	94.2	97
ASV_3	Rickettsiales-like (Anaplasmataceae)	*Litoreibacter arenae* (NR_044471)[1]	85.6	99	Uncultured clone 40C11 (JF827402)[2]	87.6	100
					Anaplasmataceae bacterium IS136 (AB190771)	87.6	100
ASV_4	Cryomorphaceae (novel)	*Parvicella tangerina* (NR_184613)	92.2	100	Uncultured clone Woods-Hole_a1725 (KF798499; Ciona intestinalis gut)	98.4	100

¹ *Litoreibacter arenae* is the single highest-scoring described species, but the subsequent hits are all Rickettsiales (Anaplasmataceae/Rickettsiaceae; e.g., *Anaplasma phagocytophilum*, Ehrlichia spp., Orientia tsutsugamushi) at ~85.2%, consistent with the Rickettsiales-like assignment.

² Highest-identity environmental match overall (unclassified); the highest taxonomically informative match is the Anaplasmataceae clone IS136 (87.6%), shown in Fig. 4.

Table S4. ASVs removed as putative contaminants, with genus assignment, SINTAX confidence and contaminant category.

ASV	Genus	Confidence	Category
ASV_1740	Corynebacterium	1	human-associated
ASV_1992	Corynebacterium	1	human-associated
ASV_1993	Corynebacterium	1	human-associated
ASV_2291	Corynebacterium	1	human-associated
ASV_3198	Corynebacterium	1	human-associated
ASV_3349	Lautropia	1	human-associated
ASV_1961	Micrococcus	1	human-associated
ASV_4057	Parasutterella	1	human-associated
ASV_2887	Staphylococcus	1	human-associated
ASV_856	Staphylococcus	1	human-associated
ASV_1549	Streptococcus	1	human-associated
ASV_2194	Streptococcus	1	human-associated
ASV_2757	Streptococcus	1	human-associated
ASV_4055	Streptococcus	1	human-associated
ASV_4060	Streptococcus	1	human-associated
ASV_4156	Streptococcus	1	human-associated
ASV_759	Streptococcus	1	human-associated
ASV_1049	Acinetobacter	1	reagent/kitome
ASV_1065	Acinetobacter	1	reagent/kitome
ASV_2183	Acinetobacter	1	reagent/kitome
ASV_263	Acinetobacter	1	reagent/kitome
ASV_2650	Acinetobacter	1	reagent/kitome
ASV_2737	Acinetobacter	1	reagent/kitome
ASV_4515	Acinetobacter	1	reagent/kitome
ASV_459	Acinetobacter	1	reagent/kitome
ASV_4646	Acinetobacter	1	reagent/kitome
ASV_725	Acinetobacter	1	reagent/kitome
ASV_919	Acinetobacter	1	reagent/kitome
ASV_948	Acinetobacter	1	reagent/kitome
ASV_882	Alcaligenes	1	reagent/kitome
ASV_3471	Bradyrhizobium	1	reagent/kitome
ASV_1199	Brevundimonas	1	reagent/kitome
ASV_1384	Brevundimonas	1	reagent/kitome
ASV_1715	Brevundimonas	1	reagent/kitome
ASV_1788	Brevundimonas	1	reagent/kitome
ASV_2535	Brevundimonas	1	reagent/kitome
ASV_4282	Brevundimonas	1	reagent/kitome
ASV_4418	Brevundimonas	1	reagent/kitome
ASV_4517	Brevundimonas	1	reagent/kitome
ASV_500	Brevundimonas	1	reagent/kitome
ASV_830	Brevundimonas	1	reagent/kitome

ASV_3512	Burkholderia	1	reagent/kitome
ASV_2414	Chryseobacterium	1	reagent/kitome
ASV_2661	Chryseobacterium	1	reagent/kitome
ASV_3630	Chryseobacterium	1	reagent/kitome
ASV_145	Methylobacterium	1	reagent/kitome
ASV_2044	Methylobacterium	1	reagent/kitome
ASV_2244	Methylobacterium	1	reagent/kitome
ASV_2818	Methylobacterium	1	reagent/kitome
ASV_3221	Methylobacterium	1	reagent/kitome
ASV_1351	Pusillimonas	1	reagent/kitome
ASV_15	Ralstonia	1	reagent/kitome
ASV_1768	Sphingomonas	1	reagent/kitome
ASV_3341	Sphingomonas	1	reagent/kitome
ASV_3635	Sphingomonas	1	reagent/kitome
ASV_318	Stenotrophomonas	1	reagent/kitome
ASV_440	Stenotrophomonas	1	reagent/kitome
ASV_1392	Limnobacter	0.99	freshwater/soil
ASV_1277	Cutibacterium	0.99	human-associated
ASV_2040	Ralstonia	0.99	reagent/kitome
ASV_690	Ralstonia	0.99	reagent/kitome
ASV_2274	Sphingomonas	0.99	reagent/kitome
ASV_2344	Hydrogenophaga	0.98	freshwater/soil
ASV_289	Stenotrophomonas	0.98	reagent/kitome
ASV_2228	Comamonas	0.97	freshwater/soil
ASV_1205	Hydrogenophaga	0.97	freshwater/soil
ASV_2381	Hydrogenophaga	0.97	freshwater/soil
ASV_74	Hydrogenophaga	0.97	freshwater/soil
ASV_1573	Mitsuaria	0.97	freshwater/soil
ASV_2474	Hydrogenophaga	0.96	freshwater/soil
ASV_3074	Stenotrophomonas	0.96	reagent/kitome
ASV_507	Hydrogenophaga	0.95	freshwater/soil
ASV_1219	Comamonas	0.94	freshwater/soil
ASV_1627	Malikia	0.94	freshwater/soil
ASV_1680	Simplicispira	0.94	freshwater/soil
ASV_177	Acidovorax	0.91	freshwater/soil
ASV_2610	Hydrogenophaga	0.91	freshwater/soil
ASV_301	Acidovorax	0.89	freshwater/soil
ASV_376	Delftia	0.89	freshwater/soil
ASV_735	Sphaerotilus	0.87	freshwater/soil
ASV_2975	Variovorax	0.86	freshwater/soil
ASV_3170	Hydrogenophaga	0.84	freshwater/soil
ASV_577	Burkholderia	0.82	reagent/kitome
ASV_3624	Acidovorax	0.81	freshwater/soil
ASV_1552	Hydrogenophaga	0.81	freshwater/soil

ASV_4530	Pusillimonas	0.81	reagent/kitome
ASV_4267	Piscinibacter	0.76	freshwater/soil
ASV_291	Curvibacter	0.75	freshwater/soil
ASV_1085	Hydrogenophaga	0.72	freshwater/soil
ASV_2782	Mitsuaria	0.71	freshwater/soil
ASV_1464	Achromobacter	0.71	reagent/kitome
ASV_2139	Acidovorax	0.7	freshwater/soil
ASV_1126	Diaphorobacter	0.7	freshwater/soil
ASV_2505	Diaphorobacter	0.7	freshwater/soil
ASV_2229	Noviherbaspirillum	0.68	freshwater/soil
ASV_1478	*Escherichia*/*Shigella*	0.62	human-associated
ASV_2170	Hydrogenophaga	0.6	freshwater/soil
ASV_3069	Simplicispira	0.6	freshwater/soil
ASV_3444	Micrococcus	0.57	human-associated
ASV_3301	Achromobacter	0.56	reagent/kitome
ASV_4574	Curvibacter	0.55	freshwater/soil
ASV_2897	Acidovorax	0.53	freshwater/soil
ASV_4702	Comamonas	0.46	freshwater/soil
ASV_875	Limnohabitans	0.44	freshwater/soil
ASV_3413	Rhodoferax	0.43	freshwater/soil
ASV_4326	Parasutterella	0.37	human-associated
ASV_4257	Aquabacterium	0.34	freshwater/soil
ASV_1181	Comamonas	0.32	freshwater/soil
ASV_1690	Limnobacter	0.31	freshwater/soil
ASV_2906	Chitinimonas	0.29	freshwater/soil
ASV_431	Sphingomonas	0.26	reagent/kitome
ASV_4558	Chitinimonas	0.25	freshwater/soil
ASV_2107	Sphingomonas	0.24	reagent/kitome
ASV_2772	Acinetobacter	0.23	reagent/kitome
ASV_1683	Aquincola	0.2	freshwater/soil
ASV_4237	Aquincola	0.2	freshwater/soil
ASV_977	Chitinimonas	0.2	freshwater/soil
ASV_3547	Limnobacter	0.2	freshwater/soil
ASV_3815	Paraburkholderia	0.2	reagent/kitome
ASV_408	Chryseobacterium	0.17	reagent/kitome
ASV_2035	Sphingomonas	0.17	reagent/kitome
ASV_2620	Chitinimonas	0.16	freshwater/soil
ASV_3645	Chitinimonas	0.16	freshwater/soil
ASV_943	Chryseobacterium	0.15	reagent/kitome
ASV_3490	Caballeronia	0.14	reagent/kitome
ASV_4272	Caballeronia	0.14	reagent/kitome
ASV_4347	Chryseobacterium	0.14	reagent/kitome
ASV_4588	Chryseobacterium	0.14	reagent/kitome
ASV_3651	Sphingomonas	0.14	reagent/kitome

ASV_1262	Chitinimonas	0.13	freshwater/soil
ASV_3007	Chitinimonas	0.13	freshwater/soil
ASV_4542	Limnobacter	0.13	freshwater/soil
ASV_1705	Chryseobacterium	0.13	reagent/kitome
ASV_1949	Chryseobacterium	0.13	reagent/kitome
ASV_3741	Chryseobacterium	0.13	reagent/kitome
ASV_796	Chryseobacterium	0.12	reagent/kitome
ASV_275	Chryseobacterium	0.11	reagent/kitome
ASV_2971	Chryseobacterium	0.11	reagent/kitome
ASV_3050	Chryseobacterium	0.11	reagent/kitome
ASV_693	Chryseobacterium	0.11	reagent/kitome
ASV_3486	Streptococcus	0.1	human-associated
ASV_285	Chryseobacterium	0.1	reagent/kitome
ASV_4380	Sphingomonas	0.1	reagent/kitome
ASV_3303	Chitinimonas	0.09	freshwater/soil
ASV_1903	Streptococcus	0.09	human-associated
ASV_1830	Sphingomonas	0.09	reagent/kitome
ASV_3894	Brevundimonas	0.08	reagent/kitome
ASV_1366	Chitinimonas	0.07	freshwater/soil
ASV_4366	Hydrogenophaga	0.07	freshwater/soil
ASV_4080	Bradyrhizobium	0.07	reagent/kitome
ASV_4236	Brevundimonas	0.07	reagent/kitome
ASV_4035	Sphingomonas	0.07	reagent/kitome
ASV_1587	Caballeronia	0.06	reagent/kitome
ASV_1155	Sphingomonas	0.04	reagent/kitome
ASV_1613	Comamonas	0.03	freshwater/soil
ASV_1020	Sphingomonas	0.03	reagent/kitome
ASV_2275	Sphingomonas	0.03	reagent/kitome
ASV_2277	Sphingomonas	0.03	reagent/kitome
ASV_4001	Sphingomonas	0.03	reagent/kitome
ASV_4221	Sphingomonas	0.03	reagent/kitome
ASV_2396	Stenotrophomonas	0.03	reagent/kitome
ASV_1942	Sphingomonas	0.02	reagent/kitome
ASV_635	Sphingomonas	0.02	reagent/kitome

Genus assignments were taken from the low-threshold (0.1) SINTAX annotation, including low-confidence assignments, because the conservative annotation collapses uncertain genus-level assignments to higher ranks and would leave many contaminant ASVs unidentified. Categories follow the reference contaminant list of Salter et al. (2014): reagent/kitome, human-associated, and freshwater/soil taxa. A minor subset (0.18% of reads) matched contaminant genera only at low confidence (<0.5). *Pseudomonas* was retained rather than removed, as the genus contains genuine marine members in addition to common reagent contaminants (Section 2.6).

Table S5. Summary of statistical tests

Section	Response	Test	Statistic	df	*p*	*Note*	*p_flag*
Larvae (stage effect)	richness	Friedman	11	5	0.0514	*k=6, n=3* blocks; min attainable *p ~0*.010	ns
Larvae (stage effect)	richness	LMM (stage, 1|tank)	5.003	5,10	0.0148	arcsine-sqrt if proportion	*
Larvae (post hoc)	richness	LMM Tukey: Healthy D-veliger - Mortality d1	2.252	10	0.295	Tukey-adjusted, 15 contrasts over 6 stages	ns
Larvae (post hoc)	richness	LMM Tukey: Healthy D-veliger - Mortality d3	2.597	10	0.184	Tukey-adjusted, 15 contrasts over 6 stages	ns
Larvae (post hoc)	richness	LMM Tukey: Healthy D-veliger - Early umbo	1.205	10	0.825	Tukey-adjusted, 15 contrasts over 6 stages	ns
Larvae (post hoc)	richness	LMM Tukey: Healthy D-veliger - Large umbo	0.56	10	0.992	Tukey-adjusted, 15 contrasts over 6 stages	ns
Larvae (post hoc)	richness	LMM Tukey: Healthy D-veliger - Eyed pediveliger	-1.712	10	0.554	Tukey-adjusted, 15 contrasts over 6 stages	ns
Larvae (post hoc)	richness	LMM Tukey: Mortality d1 - Mortality d3	0.345	10	0.999	Tukey-adjusted, 15 contrasts over 6 stages	ns
Larvae (post hoc)	richness	LMM Tukey: Mortality d1 - Early umbo	-1.047	10	0.891	Tukey-adjusted, 15 contrasts over 6 stages	ns
Larvae (post hoc)	richness	LMM Tukey: Mortality d1 - Large umbo	-1.692	10	0.565	Tukey-adjusted, 15 contrasts over 6 stages	ns
Larvae (post hoc)	richness	LMM Tukey: Mortality d1 - Eyed pediveliger	-3.964	10	0.0237	Tukey-adjusted, 15 contrasts over 6 stages	*
Larvae (post hoc)	richness	LMM Tukey: Mortality d3 - Early umbo	-1.392	10	0.731	Tukey-adjusted, 15 contrasts over 6 stages	ns
Larvae (post hoc)	richness	LMM Tukey: Mortality d3 - Large umbo	-2.037	10	0.387	Tukey-adjusted, 15 contrasts over 6 stages	ns
Larvae (post hoc)	richness	LMM Tukey: Mortality d3 - Eyed pediveliger	-4.309	10	0.0142	Tukey-adjusted, 15 contrasts over 6 stages	*

Larvae (post hoc)	richness	LMM Tukey: Early umbo - Large umbo	-0.645	10	0.984	Tukey-adjusted, 15 contrasts over 6 stages	ns
Larvae (post hoc)	richness	LMM Tukey: Early umbo - Eyed pediveliger	-2.917	10	0.116	Tukey-adjusted, 15 contrasts over 6 stages	ns
Larvae (post hoc)	richness	LMM Tukey: Large umbo - Eyed pediveliger	-2.272	10	0.288	Tukey-adjusted, 15 contrasts over 6 stages	ns
Larvae (stage effect)	shannon	Friedman	9.667	5	0.0853	*k=6, n=3* blocks; min attainable *p ~0*.010	ns
Larvae (stage effect)	shannon	LMM (stage, 1|tank)	3.537	5,10	0.0422	arcsine-sqrt if proportion	*
Larvae (post hoc)	shannon	LMM Tukey: Healthy D-veliger - Mortality d1	1.832	10	0.489	Tukey-adjusted, 15 contrasts over 6 stages	ns
Larvae (post hoc)	shannon	LMM Tukey: Healthy D-veliger - Mortality d3	1.877	10	0.465	Tukey-adjusted, 15 contrasts over 6 stages	ns
Larvae (post hoc)	shannon	LMM Tukey: Healthy D-veliger - Early umbo	-1.069	10	0.883	Tukey-adjusted, 15 contrasts over 6 stages	ns
Larvae (post hoc)	shannon	LMM Tukey: Healthy D-veliger - Large umbo	-0.477	10	0.996	Tukey-adjusted, 15 contrasts over 6 stages	ns
Larvae (post hoc)	shannon	LMM Tukey: Healthy D-veliger - Eyed pediveliger	1.787	10	0.513	Tukey-adjusted, 15 contrasts over 6 stages	ns
Larvae (post hoc)	shannon	LMM Tukey: Mortality d1 - Mortality d3	0.045	10	1	Tukey-adjusted, 15 contrasts over 6 stages	ns
Larvae (post hoc)	shannon	LMM Tukey: Mortality d1 - Early umbo	-2.902	10	0.118	Tukey-adjusted, 15 contrasts over 6 stages	ns
Larvae (post hoc)	shannon	LMM Tukey: Mortality d1 - Large umbo	-2.309	10	0.274	Tukey-adjusted, 15 contrasts over 6 stages	ns
Larvae (post hoc)	shannon	LMM Tukey: Mortality d1 - Eyed pediveliger	-0.045	10	1	Tukey-adjusted, 15 contrasts over 6 stages	ns
Larvae (post hoc)	shannon	LMM Tukey: Mortality d3 - Early umbo	-2.946	10	0.111	Tukey-adjusted, 15 contrasts over 6 stages	ns

Larvae (post hoc)	shannon	LMM Tukey: Mortality d3 - Large umbo	-2.354	10	0.258	Tukey-adjusted, 15 contrasts over 6 stages	ns
Larvae (post hoc)	shannon	LMM Tukey: Mortality d3 - Eyed pediveliger	-0.09	10	1	Tukey-adjusted, 15 contrasts over 6 stages	ns
Larvae (post hoc)	shannon	LMM Tukey: Early umbo - Large umbo	0.593	10	0.989	Tukey-adjusted, 15 contrasts over 6 stages	ns
Larvae (post hoc)	shannon	LMM Tukey: Early umbo - Eyed pediveliger	2.856	10	0.127	Tukey-adjusted, 15 contrasts over 6 stages	ns
Larvae (post hoc)	shannon	LMM Tukey: Large umbo - Eyed pediveliger	2.264	10	0.291	Tukey-adjusted, 15 contrasts over 6 stages	ns
Larvae (stage effect)	*Vibrio*	Friedman	9.667	5	0.0853	*k=6, n=3* blocks; min attainable *p ~0*.010	ns
Larvae (stage effect)	*Vibrio*	LMM (stage, 1|tank)	5.291	5,12	0.0085	arcsine-sqrt if proportion; SINGULAR FIT: tank variance estimated at zero, model reduces to fixed effects	**
Larvae (post hoc)	*Vibrio*	LMM Tukey: Healthy D-veliger - Mortality d1	-2.484	10	0.216	Tukey-adjusted, 15 contrasts over 6 stages	ns
Larvae (post hoc)	*Vibrio*	LMM Tukey: Healthy D-veliger - Mortality d3	1.133	10	0.857	Tukey-adjusted, 15 contrasts over 6 stages	ns
Larvae (post hoc)	*Vibrio*	LMM Tukey: Healthy D-veliger - Early umbo	0.946	10	0.925	Tukey-adjusted, 15 contrasts over 6 stages	ns
Larvae (post hoc)	*Vibrio*	LMM Tukey: Healthy D-veliger - Large umbo	1.955	10	0.426	Tukey-adjusted, 15 contrasts over 6 stages	ns
Larvae (post hoc)	*Vibrio*	LMM Tukey: Healthy D-veliger - Eyed pediveliger	1.667	10	0.579	Tukey-adjusted, 15 contrasts over 6 stages	ns
Larvae (post hoc)	*Vibrio*	LMM Tukey: Mortality d1 - Mortality d3	3.618	10	0.0401	Tukey-adjusted, 15 contrasts over 6 stages	*
Larvae (post hoc)	*Vibrio*	LMM Tukey: Mortality d1 - Early umbo	3.43	10	0.0534	Tukey-adjusted, 15 contrasts over 6 stages	ns

Larvae (post hoc)	*Vibrio*	LMM Tukey: Mortality d1 - Large umbo	4.439	10	0.0117	Tukey-adjusted, 15 contrasts over 6 stages	*
Larvae (post hoc)	*Vibrio*	LMM Tukey: Mortality d1 - Eyed pediveliger	4.151	10	0.0179	Tukey-adjusted, 15 contrasts over 6 stages	*
Larvae (post hoc)	*Vibrio*	LMM Tukey: Mortality d3 - Early umbo	-0.187	10	1	Tukey-adjusted, 15 contrasts over 6 stages	ns
Larvae (post hoc)	*Vibrio*	LMM Tukey: Mortality d3 - Large umbo	0.822	10	0.957	Tukey-adjusted, 15 contrasts over 6 stages	ns
Larvae (post hoc)	*Vibrio*	LMM Tukey: Mortality d3 - Eyed pediveliger	0.533	10	0.993	Tukey-adjusted, 15 contrasts over 6 stages	ns
Larvae (post hoc)	*Vibrio*	LMM Tukey: Early umbo - Large umbo	1.009	10	0.905	Tukey-adjusted, 15 contrasts over 6 stages	ns
Larvae (post hoc)	*Vibrio*	LMM Tukey: Early umbo - Eyed pediveliger	0.721	10	0.975	Tukey-adjusted, 15 contrasts over 6 stages	ns
Larvae (post hoc)	*Vibrio*	LMM Tukey: Large umbo - Eyed pediveliger	-0.288	10	1	Tukey-adjusted, 15 contrasts over 6 stages	ns
Larvae (stage effect)	*Tenacibaculum*	Friedman	11.952	5	0.0354	$k=6$, $n=3$ blocks; min attainable $p \sim 0.010$	*
Larvae (stage effect)	*Tenacibaculum*	LMM (stage, 1|tank)	6.558	5,10	0.00591	arcsine-sqrt if proportion	**
Larvae (post hoc)	*Tenacibaculum*	LMM Tukey: Healthy D-veliger - Mortality d1	2.849	10	0.128	Tukey-adjusted, 15 contrasts over 6 stages	ns
Larvae (post hoc)	*Tenacibaculum*	LMM Tukey: Healthy D-veliger - Mortality d3	0.612	10	0.988	Tukey-adjusted, 15 contrasts over 6 stages	ns
Larvae (post hoc)	*Tenacibaculum*	LMM Tukey: Healthy D-veliger - Early umbo	0.871	10	0.945	Tukey-adjusted, 15 contrasts over 6 stages	ns
Larvae (post hoc)	*Tenacibaculum*	LMM Tukey: Healthy D-veliger - Large umbo	4.048	10	0.0209	Tukey-adjusted, 15 contrasts over 6 stages	*
Larvae (post hoc)	*Tenacibaculum*	LMM Tukey: Healthy D-veliger - Eyed pediveliger	4.077	10	0.02	Tukey-adjusted, 15 contrasts over 6 stages	*

Larvae (post hoc)	*Tenacibaculum*	LMM Tukey: Mortality d1 - Mortality d3	-2.236	10	0.301	Tukey-adjusted, 15 contrasts over 6 stages	ns
Larvae (post hoc)	*Tenacibaculum*	LMM Tukey: Mortality d1 - Early umbo	-1.978	10	0.415	Tukey-adjusted, 15 contrasts over 6 stages	ns
Larvae (post hoc)	*Tenacibaculum*	LMM Tukey: Mortality d1 - Large umbo	1.199	10	0.828	Tukey-adjusted, 15 contrasts over 6 stages	ns
Larvae (post hoc)	*Tenacibaculum*	LMM Tukey: Mortality d1 - Eyed pediveliger	1.228	10	0.814	Tukey-adjusted, 15 contrasts over 6 stages	ns
Larvae (post hoc)	*Tenacibaculum*	LMM Tukey: Mortality d3 - Early umbo	0.258	10	1	Tukey-adjusted, 15 contrasts over 6 stages	ns
Larvae (post hoc)	*Tenacibaculum*	LMM Tukey: Mortality d3 - Large umbo	3.436	10	0.053	Tukey-adjusted, 15 contrasts over 6 stages	ns
Larvae (post hoc)	*Tenacibaculum*	LMM Tukey: Mortality d3 - Eyed pediveliger	3.465	10	0.0507	Tukey-adjusted, 15 contrasts over 6 stages	ns
Larvae (post hoc)	*Tenacibaculum*	LMM Tukey: Early umbo - Large umbo	3.177	10	0.0783	Tukey-adjusted, 15 contrasts over 6 stages	ns
Larvae (post hoc)	*Tenacibaculum*	LMM Tukey: Early umbo - Eyed pediveliger	3.206	10	0.075	Tukey-adjusted, 15 contrasts over 6 stages	ns
Larvae (post hoc)	*Tenacibaculum*	LMM Tukey: Large umbo - Eyed pediveliger	0.029	10	1	Tukey-adjusted, 15 contrasts over 6 stages	ns
Larvae (stage effect)	ASV_4 (Cryomorphaceae)	Friedman	14.238	5	0.0142	$k=6, n=3$ blocks; min attainable $p \sim 0.010$	*
Larvae (stage effect)	ASV_4 (Cryomorphaceae)	LMM (stage, 1|tank)	19.331	5,10	7.52e-05	arcsine-sqrt if proportion	***
Larvae (post hoc)	ASV_4 (Cryomorphaceae)	LMM Tukey: Healthy D-veliger - Mortality d1	-3.946	10	0.0244	Tukey-adjusted, 15 contrasts over 6 stages	*
Larvae (post hoc)	ASV_4 (Cryomorphaceae)	LMM Tukey: Healthy D-veliger - Mortality d3	-4.867	10	0.00627	Tukey-adjusted, 15 contrasts over 6 stages	**
Larvae (post hoc)	ASV_4 (Cryomorphaceae)	LMM Tukey: Healthy D-veliger - Early umbo	1.33	10	0.764	Tukey-adjusted, 15 contrasts over 6 stages	ns

Larvae (post hoc)	ASV_4 (Cryomorphaceae)	LMM Tukey: Healthy D-veliger - Large umbo	2.086	10	0.364	Tukey-adjusted, 15 contrasts over 6 stages	ns
Larvae (post hoc)	ASV_4 (Cryomorphaceae)	LMM Tukey: Healthy D-veliger - Eyed pediveliger	2.165	10	0.33	Tukey-adjusted, 15 contrasts over 6 stages	ns
Larvae (post hoc)	ASV_4 (Cryomorphaceae)	LMM Tukey: Mortality d1 - Mortality d3	-0.921	10	0.932	Tukey-adjusted, 15 contrasts over 6 stages	ns
Larvae (post hoc)	ASV_4 (Cryomorphaceae)	LMM Tukey: Mortality d1 - Early umbo	5.276	10	0.00353	Tukey-adjusted, 15 contrasts over 6 stages	**
Larvae (post hoc)	ASV_4 (Cryomorphaceae)	LMM Tukey: Mortality d1 - Large umbo	6.032	10	0.00128	Tukey-adjusted, 15 contrasts over 6 stages	**
Larvae (post hoc)	ASV_4 (Cryomorphaceae)	LMM Tukey: Mortality d1 - Eyed pediveliger	6.111	10	0.00116	Tukey-adjusted, 15 contrasts over 6 stages	**
Larvae (post hoc)	ASV_4 (Cryomorphaceae)	LMM Tukey: Mortality d3 - Early umbo	6.197	10	0.00104	Tukey-adjusted, 15 contrasts over 6 stages	**
Larvae (post hoc)	ASV_4 (Cryomorphaceae)	LMM Tukey: Mortality d3 - Large umbo	6.953	10	0.000408	Tukey-adjusted, 15 contrasts over 6 stages	***
Larvae (post hoc)	ASV_4 (Cryomorphaceae)	LMM Tukey: Mortality d3 - Eyed pediveliger	7.033	10	0.000372	Tukey-adjusted, 15 contrasts over 6 stages	***
Larvae (post hoc)	ASV_4 (Cryomorphaceae)	LMM Tukey: Early umbo - Large umbo	0.756	10	0.969	Tukey-adjusted, 15 contrasts over 6 stages	ns
Larvae (post hoc)	ASV_4 (Cryomorphaceae)	LMM Tukey: Early umbo - Eyed pediveliger	0.835	10	0.954	Tukey-adjusted, 15 contrasts over 6 stages	ns
Larvae (post hoc)	ASV_4 (Cryomorphaceae)	LMM Tukey: Large umbo - Eyed pediveliger	0.08	10	1	Tukey-adjusted, 15 contrasts over 6 stages	ns
Larvae (stage effect)	ASV_3 (Rickettsiales-like)	Friedman	13.232	5	0.0213	*k=6, n=3* blocks; min attainable *p ~0*.010	*
Larvae (stage effect)	ASV_3 (Rickettsiales-like)	LMM (stage, 1|tank)	8.408	5,12	0.00128	arcsine-sqrt if proportion; SINGULAR FIT: tank variance estimated at	**

						zero, model reduces to fixed effects	
Larvae (post hoc)	ASV_3 (Rickettsiales-like)	LMM Tukey: Healthy D-veliger - Mortality d1	1.483	10	0.682	Tukey-adjusted, 15 contrasts over 6 stages	ns
Larvae (post hoc)	ASV_3 (Rickettsiales-like)	LMM Tukey: Healthy D-veliger - Mortality d3	1.472	10	0.688	Tukey-adjusted, 15 contrasts over 6 stages	ns
Larvae (post hoc)	ASV_3 (Rickettsiales-like)	LMM Tukey: Healthy D-veliger - Early umbo	1.452	10	0.698	Tukey-adjusted, 15 contrasts over 6 stages	ns
Larvae (post hoc)	ASV_3 (Rickettsiales-like)	LMM Tukey: Healthy D-veliger - Large umbo	-0.23	10	1	Tukey-adjusted, 15 contrasts over 6 stages	ns
Larvae (post hoc)	ASV_3 (Rickettsiales-like)	LMM Tukey: Healthy D-veliger - Eyed pediveliger	-3.81	10	0.03	Tukey-adjusted, 15 contrasts over 6 stages	*
Larvae (post hoc)	ASV_3 (Rickettsiales-like)	LMM Tukey: Mortality d1 - Mortality d3	-0.011	10	1	Tukey-adjusted, 15 contrasts over 6 stages	ns
Larvae (post hoc)	ASV_3 (Rickettsiales-like)	LMM Tukey: Mortality d1 - Early umbo	-0.03	10	1	Tukey-adjusted, 15 contrasts over 6 stages	ns
Larvae (post hoc)	ASV_3 (Rickettsiales-like)	LMM Tukey: Mortality d1 - Large umbo	-1.713	10	0.553	Tukey-adjusted, 15 contrasts over 6 stages	ns
Larvae (post hoc)	ASV_3 (Rickettsiales-like)	LMM Tukey: Mortality d1 - Eyed pediveliger	-5.293	10	0.00345	Tukey-adjusted, 15 contrasts over 6 stages	**
Larvae (post hoc)	ASV_3 (Rickettsiales-like)	LMM Tukey: Mortality d3 - Early umbo	-0.019	10	1	Tukey-adjusted, 15 contrasts over 6 stages	ns
Larvae (post hoc)	ASV_3 (Rickettsiales-like)	LMM Tukey: Mortality d3 - Large umbo	-1.702	10	0.559	Tukey-adjusted, 15 contrasts over 6 stages	ns
Larvae (post hoc)	ASV_3 (Rickettsiales-like)	LMM Tukey: Mortality d3 - Eyed pediveliger	-5.282	10	0.0035	Tukey-adjusted, 15 contrasts over 6 stages	**
Larvae (post hoc)	ASV_3 (Rickettsiales-like)	LMM Tukey: Early umbo - Large umbo	-1.683	10	0.57	Tukey-adjusted, 15 contrasts over 6 stages	ns
Larvae (post hoc)	ASV_3 (Rickettsiales-like)	LMM Tukey: Early umbo - Eyed pediveliger	-5.262	10	0.00359	Tukey-adjusted, 15 contrasts over 6 stages	**

Larvae (post hoc)	ASV_3 (Rickettsiales-like)	LMM Tukey: Large umbo - Eyed pediveliger	-3.58	10	0.0425	Tukey-adjusted, 15 contrasts over 6 stages	*
PERMANOVA	Bray-Curtis (larvae)	adonis2 ~ stage, blocks=tank	5.704	5,12	0.001	R^2 = 0.704; permutations restricted within tank	**
PERMANOVA	Bray-Curtis (larvae)	betadisper (homogeneity)	1.433	5,12	0.293	a non-significant result supports the PERMANOVA as a location effect rather than a dispersion effect	ns
Water types (all 6 groups)	shannon	Kruskal-Wallis	16.392	5	0.00581	*n=3* per group	**
Water: Sand-filtered vs EQ(pooled)	shannon	Mann-Whitney (exact)	27	-	0.00909	*n=3* vs *n=9;* EQ pools three months, which differ substantially	**
Water: Sand-filtered vs PEC	shannon	Mann-Whitney (exact)	9	-	0.1	*n=3* vs *n=3:* two-sided *p ca*nnot fall below 0.10	ns
Water types (all 6 groups)	*Tenacibaculum*	Kruskal-Wallis	14.661	5	0.0119	*n=3* per group	*
Water: Sand-filtered vs EQ(pooled)	*Tenacibaculum*	Mann-Whitney (exact)	27	-	0.00909	*n=3* vs *n=9;* EQ pools three months, which differ substantially	**
Water: Sand-filtered vs PEC	*Tenacibaculum*	Mann-Whitney (exact)	9	-	0.1	*n=3* vs *n=3:* two-sided *p ca*nnot fall below 0.10	ns
Water types (all 6 groups)	*Vibrio*	Kruskal-Wallis	15.854	5	0.00727	*n=3* per group	**
Water: Sand-filtered vs EQ(pooled)	*Vibrio*	Mann-Whitney (exact)	1	-	0.0182	*n=3* vs *n=9;* EQ pools three months, which differ substantially	*
Water: Sand-filtered vs PEC	*Vibrio*	Mann-Whitney (exact)	0	-	0.1	*n=3* vs *n=3:* two-sided *p ca*nnot fall below 0.10	ns
Water types (all 6 groups)	*Thiomicrorhabdus*	Kruskal-Wallis	15.489	5	0.00846	*n=3* per group	**
Water: Sand-filtered vs EQ(pooled)	*Thiomicrorhabdus*	Mann-Whitney (exact)	0	-	0.00909	*n=3* vs *n=9;* EQ pools three months, which differ	**

						substantially; ties present, exact conditional test used	
Water: Sand-filtered vs PEC	*Thiomicrorhabdus*	Mann-Whitney (exact)	0	-	0.1	*n=3* vs *n=3:* two-sided *p ca*nnot fall below 0.10; ties present, exact conditional test used	ns

Larvae. Six developmental stages were sampled from each of three rearing tanks (n = 3 per stage). Stage effects were tested with linear mixed models (tank as a random intercept) on untransformed diversity indices and on arcsine-square-root transformed relative abundances, with Friedman tests as a non-parametric check. Pairwise contrasts were Tukey-adjusted across all 15 comparisons among the six stages. For *Vibrio* and ASV_3 the tank variance was estimated at the boundary (singular fit); the random intercept is effectively absent and the model reduces to a fixed-effects analysis, which accounts for the differing denominator degrees of freedom between the stage effect and the pairwise contrasts. ASV_3 was undetected in nine of the eighteen larval samples, so the mixed model for this taxon should be read alongside the Friedman result.

Community structure. PERMANOVA was performed on Bray–Curtis distances with 999 permutations restricted within tank. Free permutation gave an identical result (F = 5.704, R^2 = 0.704, p = 0.001), indicating that the outcome is not sensitive to the permutation scheme. The non-significant betadisper result supports interpretation of the PERMANOVA as a location rather than a dispersion effect.

Water. With n = 3 per water type, the two-sided Mann–Whitney p cannot fall below 0.10, so no 3 vs 3 comparison can attain significance regardless of effect size. EQ samples from three months were therefore pooled (n = 9) for comparison with sand-filtered water; the three months differ substantially from one another (see monthly means), and this comparison should be read together with the effect sizes rather than the p-value alone. All Mann–Whitney tests are exact conditional tests, which is necessary where tied values are present.

The contrast between healthy D-veligers and day 1 of mortality for *Vibrio* was specified in advance as the primary test of the study and is reported unadjusted in the main text (p = 0.033); the Tukey-adjusted value is given here.
